# Disruption of hypoxia-inducible fatty acid binding protein 7 induces beige fat-like differentiation and thermogenesis in breast cancer cells

**DOI:** 10.1186/s40170-020-00219-4

**Published:** 2020-07-06

**Authors:** Masahiro Kawashima, Karim Bensaad, Christos E. Zois, Alessandro Barberis, Esther Bridges, Simon Wigfield, Christoffer Lagerholm, Ruslan I. Dmitriev, Mariko Tokiwa, Masakazu Toi, Dmitri B. Papkovsky, Francesca M. Buffa, Adrian L. Harris

**Affiliations:** 1 Department of Oncology, Molecular Oncology Laboratories, Weatherall Institute of Molecular Medicine, University of Oxford, John Radcliffe Hospital, Oxford, OX3 9DS UK; 2 Department of Oncology, Computational Biology and Integrative Genomics Lab, CRUK/MRC Institute for Radiation Oncology, University of Oxford, Old Road Campus Research Building, Roosvelt Drive, Oxford, OX3 7DQ UK; 3 Wolfson Imaging Centre, Weatherall Institute of Molecular Medicine, University of Oxford, John Radcliffe Hospital, Oxford, OX3 9DS UK; 4 School of Biochemistry and Cell Biology, University College Cork, Cavanagh Pharmacy Building, 1.28, College Road, Cork, Ireland; 5 Department of Breast Surgery, Graduate School of Medicine, Kyoto University, 54 Shogoin-Kawahara-cho, Sakyo-ku, Kyoto, 606 8507 Japan; 6 Institute for Regenerative Medicine, I.M. Sechenov First Moscow State University, Moscow, Russian Federation

**Keywords:** Hypoxia, Fatty acid, Thermogenesis, FLIM, Modification of radiation sensitivity, Breast cancer, UCP1

## Abstract

**Background:**

Humans produce heat through non-shivering thermogenesis, a metabolic process that occurs in inducible beige adipocytes expressing uncoupling protein 1 (UCP1). UCP1 dissipates the proton gradient of the mitochondrial inner membrane and converts that energy into heat. It is unclear whether cancer cells can exhibit autonomous thermogenesis. Previously, we found that the knockdown of hypoxia-inducible fatty acid binding protein 7 (FABP7) increased reactive oxygen species (ROS) in breast cancer cells. ROS are known to induce beige adipocyte differentiation.

**Methods:**

We investigated the association of tumor hypoxia, FABP7, and UCP1 across breast cancer patients using METABRIC and TCGA data sets. Furthermore, using a breast cancer cell line, HCC1806, we tested the effect of FABP7 knockdown on cellular physiology including thermogenesis.

**Results:**

We found a strong mutual exclusivity of FABP7 and UCP1 expression both in METABRIC and in TCGA, indicating major metabolic phenotypic differences. FABP7 was preferentially distributed in poorly differentiated-, estrogen receptor (ER) negative tumors. In contrast, UCP1 was highly expressed in normal ducts and well-differentiated-, ER positive-, less hypoxic tumors. In the cell line-based experiments, UCP1 and its transcriptional regulators were upregulated upon FABP7 knockdown. UCP1 was induced in about 20% of cancer cells, and the effect was increased further in hypoxia. UCP1 depolarized mitochondrial membranes at the site of expression. UCP1 induction was associated with the increase in proton leak, glycolysis, and maximal respiration, mimicking the typical energy profile of beige adipocytes. Most importantly, UCP1 induction elevated cancer cell temperature associated with increased vulnerability to hypoxia and γ-irradiation.

**Conclusions:**

We demonstrated that breast cancer cells can undergo thermogenesis through UCP1 induction. Disrupting FABP7-mediated fatty acid metabolism can unlock UCP1-mediated thermogenesis, potentially making it possible to develop therapies to target thermogenesis. Further study would be warranted to investigate the effect of rise in temperature of cancer cells on patients’ outcomes and the relationship to other metabolic pathways.

## Background

Heat regulates multiple physiological processes by affecting cellular metabolism. Metabolic alteration has been noted as a key hallmark of cancer biology [1]. Despite the fact that heat influences cellular metabolism, few studies have investigated how heat production influences cancer physiology. Classical studies on human breast cancer suggested that elevated angiogenesis and increased blood flow caused higher temperatures in cancer tissue [2–4]. However, our recent study on colon cancer cells described the tissue gradient of cellular temperature in a tumor spheroid model: the cellular temperatures in the hypoxic core of spheroids were higher than on the surface, and this difference was not observed in 2D culture systems [5]. This study suggested that cancer cells may have the ability to generate heat by themselves and vary their temperature in response to oxygen or nutrient availability.

Evolutionally, human beings faced the demand to increase their body temperature to survive in harsh cold environments. As a result, they gained two different ways of thermogenesis: shivering and non-shivering thermogenesis [6]. In shivering thermogenesis, energy released from muscle contractions produces heat, whereas in non-shivering thermogenesis, brown adipocytes are the main source of heat production. Brown adipocytes specifically express uncoupling protein 1 (UCP1). UCP1 is a mitochondrial carrier protein that uncouples the association between complex V with the electron transport chain (ETC), dissipating the proton gradient across the inner mitochondrial membrane. This uncoupling of complex V from ETC results in the generation of heat instead of ATP [7].

Although human neonates have abundant brown fat, adults lose most of it. Instead, they appear to possess beige adipocytes, with morphological and functional resemblance to brown adipocytes [8]. Beige adipocytes appear in white fat as small deposits after specific stimulation and generate heat through UCP1 induction. Unlike with brown adipocytes, beige adipocytes require inducers for differentiation and activation [9]. Cold exposure generates beige adipocytes through upregulating transcriptional regulators PR/SET domain 16 (PRDM16), peroxisome proliferator-activated receptor gamma coactivator 1-alpha (PGC1α), and their downstream targets including UCP1. In addition, cold temperatures elevate cyclic AMP (cAMP) to maximize UCP1 activity in beige adipocytes [10]. Reactive oxygen species (ROS) are also key inducers of beige fat differentiation [11, 12]. Beige adipocytes compensate for UCP1-mediated energy expenditure through increasing mitochondrial respiration, specifically elevating glycolysis and pyruvate production [13].

Previously, we demonstrated that a protein involved in fatty acid transport, fatty acid binding protein 7 (FABP7) experienced HIF1-dependent upregulation in hypoxic breast cancer cells [14]. FABP7 preferentially binds to polyunsaturated fatty acids (PUFAs), and its knockdown led to increased ROS in cancer cells under hypoxia and hypoxia-reoxygenation [14, 15], potentially due to loss of its PUFA-scavenging function. In the current study, we investigate whether FABP7 inhibition results in another metabolic outcome in breast cancer. We show that FABP7 knockdown upregulated the genes related to beige fat differentiation in a breast cancer cell line. The cancer cells with FABP7 knockdown expressed high level of UCP1 and increased cellular temperature. These phenomena were associated with decreased cell growth and increased sensitivity to oxidative stress. Considering heat can enhance tumor immunity through modulating cytotoxic activity and tissue penetration of immune cells [16, 17], FABP7 inhibition could be an attractive target for cancer therapy with direct effects on tumor growth and potentially with indirect effects on the tumor temperature and immune response. Several inhibitors of fatty acid transport and desaturation have been developed to combat metabolic diseases [18–20], and their effect for cancer should be investigated in combinations in the future.

## Methods

### Cell culture

Cells were obtained from the American Type Culture Collection and maintained in a humidified incubator at 5% CO_2_ and 37 °C. For hypoxic exposure, cells were grown in an INVIVO_2_ 400 hypoxic workstation (Baker Ruskinn) using a continuous flow of a humidified mixture of 0.1% O_2_, 5% CO_2_, and 94.9% N_2_. Cells were maintained in Dulbecco’s modified Eagle’s medium (10 mM glucose) (Gibco) supplemented with 10% fetal bovine serum (FBS).

### Gene silencing by RNA interference

The lentiviral transduction particles containing a FABP7 shRNA expression cassette (Mission® shRNA, TRCN0000059744) or a non-targeting shRNA sequence (SHC002U) were purchased from Sigma-Aldrich. Cells were transduced with a MOI of 3, in the presence of 6 μg/ml polybrene (Sigma Aldrich) for 24 h. Cells expressing the shRNA were selected in puromycin (Invitrogen)-containing medium (2 μg/ml). After the selection, cells were suspended in FBS containing 5% DMSO (v/v) and stored at − 80 °C. In a set of experiments, cells were refreshed in every 1 month.

### Lipid peroxidation assay

Cellular lipid peroxidation levels were measured using Image-iT® Lipid Peroxidation Kit (Thermo Fisher) according to the manufacturer’s instructions. Cells were exposed to hypoxia for 24 h or 4 Gy of ionizing radiation. The fluorescence was measured with an Attune NxT Flow Cytometer (Thermo Fisher) using two different filter sets: the one at excitation/emission of 488/530 nm for detecting oxidized lipids and the other at excitation/emission of 561/620 nm for detecting reduced lipids. Lipid peroxidation levels were calculated as a ratio of the intensity of green (530 nm) fluorescence to that of red (620 nm) fluorescence.

### Cell-cycle analysis

To evaluate cell-cycle distribution, cells were washed with ice-cold PBS, resuspended in 1 ml of PBS, and stored after the dropwise addition of 3 ml of ice-cold 70% ethanol at 4 °C until analysis. Cells were washed twice with ice-cold PBS and stained with a PI solution (100 mg/ml) (Sigma Aldrich) containing DNase-free RNase (12 mg/ml) and 1% of Triton X100. After overnight incubation at 4 °C, cells were analyzed with Attune NxT Flow Cytometer using a filter set at excitation/emission of 488/590 nm.

### Quantitative PCR

RNA was isolated using TRIZOL® Reagent (Invitrogen), and complementary DNA was generated from the RNA using High Capacity cDNA Reverse Transcription Kit (Applied Biosystems), according to the manufacturers’ instructions. Real-time PCR was performed on a 7900HT Fast Real Time PCR System (Applied Biosystems) using the SensiMix^TM^ SYBR No-Rox kit (Bioline). The comparative threshold cycle method was used to present the relative gene expressions. Expression data were normalized to the expression of the two control genes: ACTB and HPRT1. Primer sequences were as follows: ACTB forward: ATTGGCAATGAGCGGTTC; ACTB reverse: GGATGCCACAGGACTCCAT; HPRT1 forward: CCAGTCAACAGGGGACATAAA; HPRT1 reverse: CACAATCAAGACATTCTTTCCAGT; FABP7 forward: TGAAACCACTGCAGATGATAGAA; FABP7 reverse: TTTCTTTGCCATCCCATTTC; PRDM16 forward: ATGGGAGCAAATACTGACGG; PRDM16 reverse: CACGCAGAACTTCTCACTGC; PGC-1α forward: GCCAAACCAACAACTTTATCTCTTC; PGC-1α reverse: CACACTTAAGGTGCGTTCAATAGTC; UCP1 forward: TCTACGACACGGTCCAGG; UCP1 reverse: GTCTGACTTTCACGACCTCTG.

### Western blots

Cell were lysed in RIPA buffer supplemented with cOmplete® Protease Inhibitor Cocktail (Roche) and PhosSTOP® Phosphatase Inhibitor Cocktail (Roche). The lysates were centrifuged at 20,000*g* at 4 °C for 15 min, and the supernatants were incubated with DTT (100 mM) and NuPAGE® LDS Sample Buffer (Invitrogen) at 70 °C for 10 min. Proteins were separated on Novex® 4–12% Tris-Glycine Mini Gels (Invitrogen) and transferred to a PVDF membrane. The membrane was incubated with 5% skim milk at room temperature for 1 hr and subsequently with primary antibodies at 4 °C for overnight. For the detection of FABP7, the step of centrifuging cell lysates was omitted, and 5% BSA was used as a blocking solution. Primary antibodies were as follows and used at 1:1000 dilution unless otherwise stated: rabbit anti-FABP7 (#13347, Cell Signaling Technology), rabbit anti-UCP1 (U6382, Sigma Aldrich), rabbit anti-PGC-1α (1:200 v/v) (sc-13067, Santa Cruz Biotechnology), rabbit anti-PRDM16 (1:500 v/v) (ab106410, Abcam), rabbit anti-CREB (#9197, Cell Signaling Technology), and rabbit anti-phospho-CREB (#9198, Cell Signaling Technology). Appropriate secondary horseradish peroxidase-linked antibodies were used (Dako, UK). Immunoreactivity was detected with ECL Prime Western Blotting Detection Reagent (Amersham) and visualized using ImageQuant LAS 4000 mini (GE Healthcare).

### Immunofluorescence

Cells were grown on cover slips and fixed with 4% paraformaldehyde at room temperature for 10 min. For the visualization of polarized mitochondria, cells were incubated with 150 nM of Mito Tracker® Red CMXRos (Molecular Probes) at 37 °C for 30 min prior to the fixation. Cells were permeabilized with 0.1% Triton X-100 for 5 min and then blocked in 5% normal horse serum for 30 min. They were incubated with rabbit anti-UCP1 diluted in blocking solution (1:500 v/v) (U6382, Sigma Aldrich) overnight at 4 °C, labeled with a secondary antibody conjugated with Alexa® 488 (Invitrogen) at room temperature for 30 min, and mounted with ProLong® Diamond Antifade Mountant with DAPI (Molecular Probes). For calculating UCP1-positive cell proportion, at least 6 images were acquired in each condition with a Delta Vision Elite High Resolution Microscope (GE Healthcare Life Science). For analyzing the colocalization of UCP1 and Mito Tracker, images were acquired with a Zeiss LSM 780 confocal microscope (Carl Zeiss) and reconstituted with ImageJ 1.51 g (National Institutes of Health).

### Assessment of mitochondrial membrane potential

Mitochondrial membrane potential was analyzed with BD^TM^ MitoScreen Kit (BD Bioscience) according to the manufacturer’s instructions. Cells were stained with JC-1 solution for 30 min and analyzed with an Attune NxT Flow Cytometer using two different filter sets: the one at excitation/emission of 488/530 nm for detecting polarized mitochondria and the other at excitation/emission of 561/585 nm for detecting depolarized mitochondria.

### Assessment of mitochondrial respiration and cellular glycolytic function

Seahorse Cell Mito Stress Test Kit (Agilent) and Seahorse Glycolysis Stress Test Kit (Agilent) were used to assess mitochondrial respiration and cellular glycolytic function, respectively. Cells were plated in a 96-well Seahorse XF Cell Culture Microplate (40,000 cells/well) 1 day prior to the assay with normal growth media. For Mito Stress Test, the growth media was replaced to Seahorse Base Media (Agilent) supplemented with 10 mM glucose, 4 mM glutamine, and 1 mM sodium pyruvate (pH 7.4 at 37 °C), and the cells were transferred to non-CO_2_ incubator (37 °C) 1 hr prior to the assay. In the assay, 0.5 μM oligomycin, 1 μM FCCP, and 0.5 μM rotenone/antimycin A were sequentially injected, and oxygen consumption rate (OCR) was monitored using Seahorse XF^e^96 Extracellular Flux Analyzer (Seahorse Bioscience). For Glycolysis Stress Test, the growth media was replaced to Seahorse Base Media (Agilent) supplemented with 4 mM glutamine and 1 mM sodium pyruvate (pH 7.35 at 37 °C) for the preconditioning. In the assay, 10 mM glucose, 1 μM oligomycin, and 50 mM 2-deoxy-glucose were sequentially injected, and extra cellular acidification rate (ECAR) was monitored. After the assays, the OCR and ECAR were normalized with the relative fluorescent intensities from CyQUANT® Cell Proliferation Assay Kit. All the parameters were generated and analyzed on a Seahorse XF report generator.

### Measurement of cellular temperature

For the measurement of cellular temperature, the temperature-sensitive fluorescent nanoprobe (T probe) was used as previously described [5]. Cells were grown in μ-Slide 8 Well Glass Bottom (ibidi) and then incubated with the growth media containing 1.5 μg/ml of T probe for 16 h. After washing twice with growth media, fluorescence lifetime imaging microscopy (FLIM) was performed on Leica SP8X Inverted Confocal/Gated STED microscope (Leica microsystems) equipped with a thermal control chamber and an objective lens (HC PL APO 63 × /1.20 W with Motorized Correction Collar, Leica microsystems) under the continuous flow of 5% CO_2_. T probe was excited with a tunable pulsed white laser (561 nm, 40 Hz), and its emission was collected at 570–620 nm with 50 times of repetitions. Fluorescence lifetimes were calculated by monoexponential decay fitting (2.5–15 ns). Calibration curve of T probe was generated by collecting fluorescence lifetimes of stained cells at 3 different incubator temperatures: 32 °C, 37 °C, and 42 °C. After generating the calibration curve, fluorescence lifetimes in cells with/without the specific knockdown were collected at 37 °C of incubator temperature. Cells were equilibrated for at least 30 min at desired temperatures prior to measurements.

### Cell proliferation assay

Cells were seeded in 96-well plate at a density of 1000 cells/well and exposed to hypoxia or ionizing radiation. Cell number was determined using CyQUANT® Cell Proliferation Assay Kit (Molecular Probes) according to the manufacturer's instructions. Fluorescence was measured with a SpectraMax microplate reader (Molecular Devices) with excitation at 485 nm and emission detection at 530 nm.

### Clonogenic assay

Cells were seeded in 100-mm dish at a density of 125 cells/dish, and, 24 h later, they were exposed to hypoxia or ionizing radiation. For hypoxia experiments, cells were cultured under hypoxia for 72 h and then placed back to normoxia. Cells were allowed to grow for 14–15 days until colonies became visible and clear. Colonies were fixed with acetic acid/methanol solution (1:7 v/v) for 5 min, stained with 0.5% crystal violet solution for 2 h, and rinsed with tap water. Size and number of colonies were measured using a ColCount automated colony counter (Optronix). Plating efficacy (PE) and surviving fraction (SF) were calculated from the following equations: (PE) = (number of colonies formed/number of cells seeded) × 100 (%) and (SF) = (number of colonies formed after irradiation)/(number of cells seeded × PE) [21]. For radiation experiments, cells were irradiated 24 h after seeding with the IBL 637 Cesium-137 *γ*-ray machine (The dose rate was 0.0485 Gy/s).

### Immunohistochemistry

Breast cancer primary tumor and the paired normal mammary glands were collected through partial or total mastectomy at the Department of Breast Surgery, Kyoto University Hospital. Written informed consent was obtained from all patients prior to sample collection. The study protocol was approved by the Ethics Committee for Clinical Research, Kyoto University Hospital (authorization number G424). The sections were incubated with citrate buffer at 120 °C for 5 min and with 3% hydrogen peroxide/methanol solution for 30 min and then blocked in PBS containing 5% normal goat serum and 1% bovine serum albumin for 10 min. They were incubated with rabbit anti-UCP1 diluted in blocking solution (1:500 v/v) (U6382, Sigma Aldrich) overnight at 4 °C. Staining was performed using ENVISION+HRP (DAKO) and DAB+ (DAKO) according to the manufacturer’s instructions. Sections were counterstained with Mayer’s hematoxylin solution and imaged using an optical microscope (BZ-9000, Keyence, Osaka, Japan). UCP1 expression was scored as “negative/weak,” “moderate,” and “strong” by 2 independent evaluators. The association between UCP1 expression and clinicopathological features was assessed using *χ*-square test.

### Gene expression analysis and survival analysis using breast cancer cohorts

Gene expression and clinical data for both Molecular Taxonomy of Breast Cancer International Consortium (METABRIC) [22] and The Cancer Genome Atlas (TCGA) [23] breast studies were downloaded from the cBioPortal for cancer genomics at https://www.cbioportal.org. We considered microarray data for METABRIC and RNA Seq V2 RSEM normalized gene expression data for TCGA. Genes with NA values in more than half of the samples were filtered out as a pre-processing step. Likewise, we removed samples with NA values in more than half of the genes. Sequencing data was then managed by the transformation log_2_ (*x* + 1), where *x* stands for the original expression value. The gene signature as defined previously was used to investigate the extent of hypoxia of METABRIC and TCGA samples [24]. The analysis was performed by using the R software (https://www.r-project.org/). In particular, we used the sigQC R package [25] to understand if the properties of such previously identified gene signature were conserved on the above-mentioned datasets. After confirming that we could use the signature, we focused on two measures of signature summary provided by sigQC (i.e., the median score and the gene set enrichment score computed via the single-sample Gene Set Enrichment Analysis (ssGSEA) algorithm) for further elaboration. The correlation between the hypoxia scores and the genes of interest (UCP1 and FABP7) was computed as Spearman’s rank correlation and reported on scatterplots (ggpubr package). To investigate the association between the patients’ survival time and different covariates (i.e., hypoxia, UCP1, and FABP7 expressions), we used the Cox proportional-hazards model (survival package [26]). We then took advantage of the Kaplan-Meier (KM) method to estimate the survival probability from observed survival times (survival package). Since we wanted to display how estimated survival depends upon the UCP1 and FABP7 values (low/high), we divided the samples in groups by using two different methods, i.e., the median and then *k*-means, *k* = 2 (stats package). In this paper, we show the result using *k*-means as a representative since we found that both methods showed similar results.

### Statistical analyses

Statistical analysis of numerical data and generation of graphs was carried out on Prism 6.0 (GraphPad) using unpaired Student’s *t* tests. All results are represented with means ± SD unless otherwise stated. Significance of difference is represented by *p < 0.05, **p < 0.01, ***p < 0.001, and ****p < 0.001.

## Results

### Mutual exclusivity between FABP7 and UCP1 in human breast cancers

**Fig. 1:**
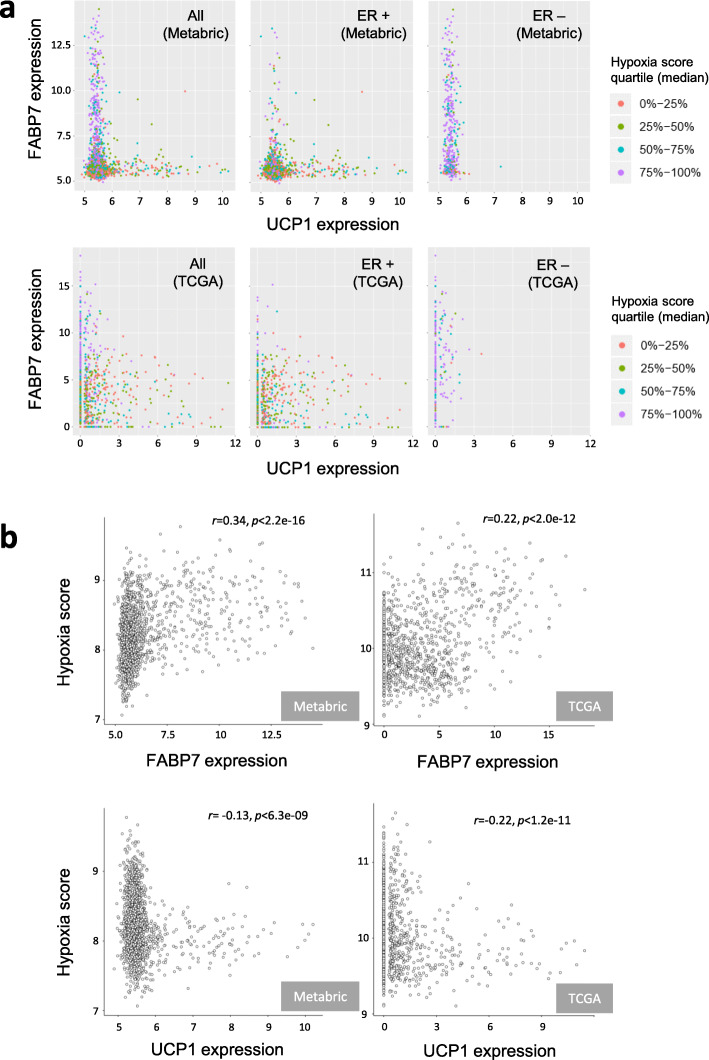
Mutual exclusivity between FABP7 and UCP1 expression in primary breast cancer tissues. **a** Association of FABP7, UCP1, and hypoxia median score in the METABRIC (*n* = 1904, upper panels) and TCGA (*n* = 960, lower panels) breast cancer cohorts. X and y axes show UCP1 and FABP7 expression, respectively. Hypoxia median score quartiles are indicated using different colors (orange, green, blue, and purple). **b** Correlation analyses of hypoxia score with FABP7 (upper panels) and UCP1 expression (lower panels)

We found a strong mutual exclusivity between FABP7 and UCP1 expression in the METABRIC and TCGA breast cancer cohorts (Fig. 1a). High FABP7 expression was limited to hypoxic estrogen receptor (ER) negative tumous, whereas UCP1 expression was preferentially observed in the less-hypoxic ER positive tumors (Fig. 1a and S1a). As a result, FABP7 and UCP1 expression exhibited mutually exclusive distribution across the patients. Similarly, a negative correlation between UCP1 expression and tumor hypoxia was confirmed in the analyses using two different scores (refer to the “Materials and Methods” section for details) (Fig. 1b and S1b). In contrast, the effect of hypoxia on FABP7 expression was reciprocal between two scores (Fig. 1b and S1b).

**Fig. 2:**
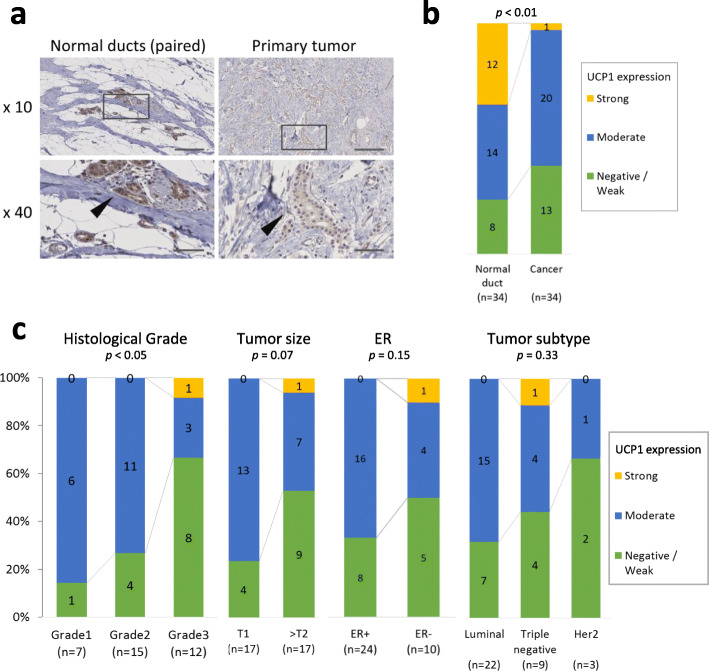
Association of UCP1 expression with clinicopathological features of breast cancer patients. **a** Representative immunohistochemical images of UCP1 expression in normal mammary ducts and paired invasive cancer. Images in the gray boxes are displayed in the lower row with higher magnification. The arrow heads indicate ductal cells and cancer cells. Scale bars; 200 μm (upper row) and 50 μm (lower row). **b** Immunohistochemistry of UCP1 expression patterns in normal mammary grands and paired invasive cancer cells (*n* = 34). **c** Immunohistochemistry of UCP1 expression in invasive cancer cells with histological grade, tumor size, estrogen receptor (ER) status, and tumor subtypes (*n* = 34). Digits in the bars represent number of cases. The vertical axis represents proportion of cases with a given characteristic to sum of all cases (%)

By immunohistochemistry (IHC), we found that UCP1 was highly expressed in normal mammary duct epithelium compared to the paired invasive cancer cells (Fig. 2a, b). In the comparison across the invasive cancer cells, higher-grade tumors had significantly lower UCP1 expression (Fig. 2c). In addition, UCP1 expression tended to be lower in large-sized and ER negative tumors although it was not statistically significant (Fig. 2c).

The gene expression analyses and IHC findings suggested that there were major phenotypic differences between FABP7 high- and UCP1 high-tumors, namely, UCP1 high-tumors were less hypoxic, ER positive, and well-differentiated compared to FABP7 high-tumors.

### FABP7 knockdown induced UCP1 expression

**Fig. 3:**
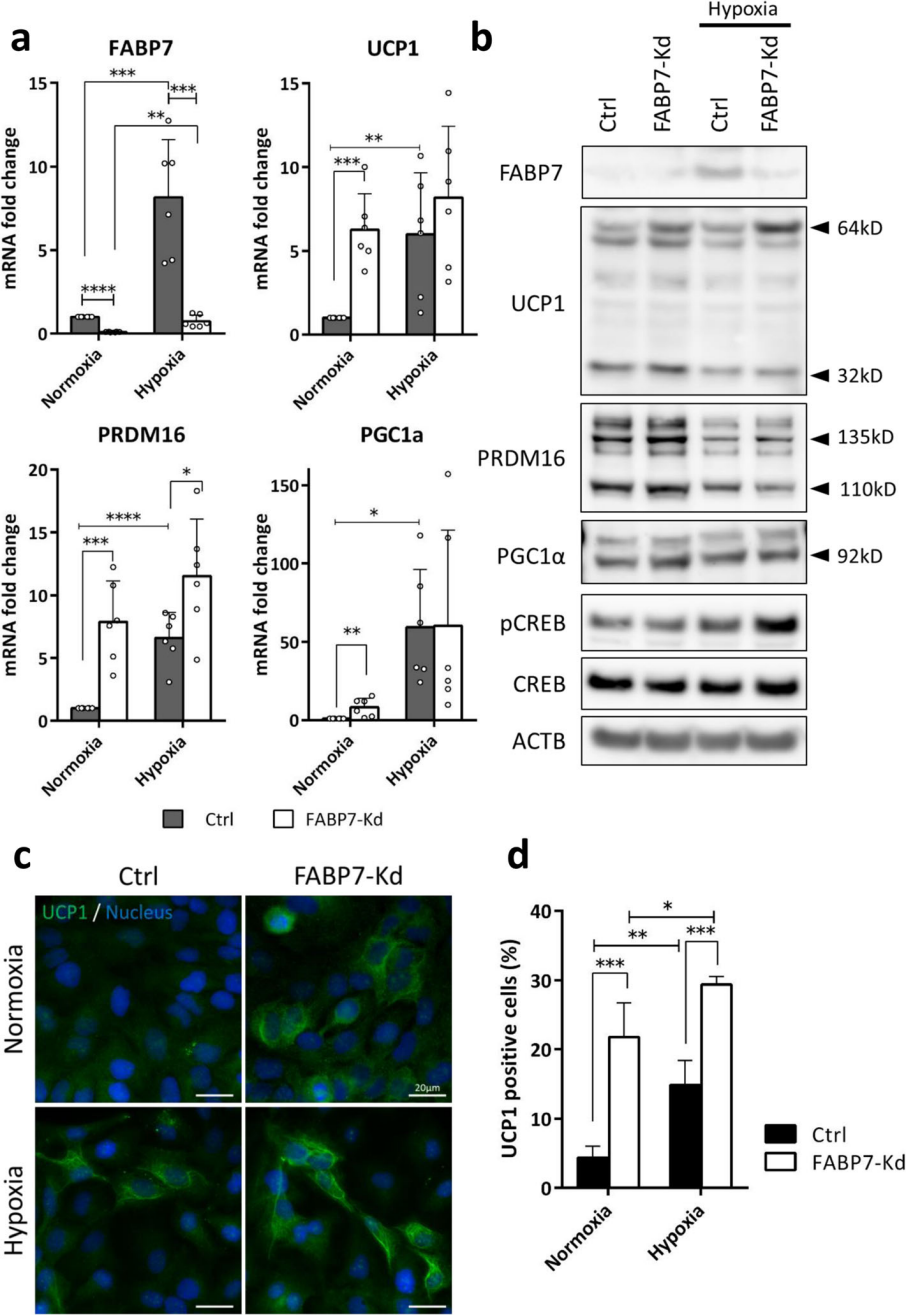
FABP7 knockdown (FABP7-Kd) and hypoxic exposure induced UCP1 expression in cancer cells. **a** FABP7, PRDM16, PGC-1α, and UCP1 expression in controls (Ctrl) and FABP7-Kd after 48 h normoxia or hypoxia (0.1% O_2_). **b** Representative western blots of FABP7, UCP1, PRDM16, PGC-1α, phosphorylated CREB (pCREB), and CREB after 48 h normoxia or hypoxia (0.1% O_2_). UCP1 bands appeared at 32 kD (monomer) and 64 kD (dimer). Four different PRDM16 isoforms were detected. **c** UCP1 expression (green) in cells cultured under normoxia or hypoxia (0.1% O_2_) for 48 h. Nuclei were stained with DAPI (blue). Scale bars; 20 μm. **d** Proportion of UCP1-expressing cells in Ctrl and FABP7-Kd after 48 h normoxia or hypoxia (0.1% O_2_). Error bars, SD; *p < 0.05, **p < 0.01, ***p < 0.001; *n* = 3

The mutually exclusive relationship between FABP7 and UCP1 expression suggested that FABP7 could negatively regulate UCP1-mediated thermogenesis in breast cancer. Therefore, we tested whether FABP7 knockdown could affect the differentiation status of breast cancer cells and UCP1-mediated thermogenesis. FABP7 knockdown increased transcription and translation of UCP1 and its master regulators (PRDM16 and PGC1α) under normoxia (Fig. 3a, b). Hypoxic exposure (0.1% O_2_, 48 h) upregulated UCP1 transcription. However, we found no difference in UCP1 transcription between FABP7-knockdown cells and controls in hypoxia (Fig. 3a). Instead,, the dimeric, presumably active form of UCP1 increased in FABP7-knockdown cells under hypoxia, indicating that the combination of FABP7 knockdown and low-oxygen conditions maximized the protein’s activity (Fig. 3b). Hypoxia upregulated PRDM16 and PGC1α transcription (Fig. 3a), but this did not result in any proportionate change in protein (Fig. 3b). Instead, we observed an increase in phosphorylated cAMP responsive element binding protein (pCREB), a protein involved in increasing UCP1 activity in beige adipocytes, in hypoxic FABP7-knockdown cells (Fig. 3b). Thus, pCREB may have contributed for the further activation of UCP1 in hypoxia.

IHC revealed that UCP1 protein expressed in a HCC1806 subpopulation (Fig. 3c). Among FABP7-knockdown cells, 21 ± 5% expressed UCP1, whereas only 4 ± 1.7% expressed UCP1 among control cells (*p* = 0.004) (Fig. 3d). Hypoxic exposure induced UCP1 expression in 15 ± 3.5% of control cells, whereas it induced UCP1 in 29 ± 1.2% of FABP7-knockdown cells (*p* = 0.002) (Fig. 3d). To exclude the non-specific bindings of the anti-UCP1 antibody, we verified its specificity using recombinant UCP1 peptide (ab24282, Abcam). The UCP1 staining has completely disappeared after the treatment of the recombinant peptide, which confirmed the specificity of the antibody (Fig. S2a for western blot and Fig S2b for IHC). Collectively, these results show that FABP7 knockdown upregulates UCP1 in the breast cancer cells presumably by inducing beige fat-like differentiation under normoxia. In addition, FABP7 knockdown maximized UCP1 induction under hypoxia presumably through the increase of pCREB.

### FABP7 knockdown enhanced mitochondrial proton leak and metabolic rate of cancer cells

**Fig. 4:**
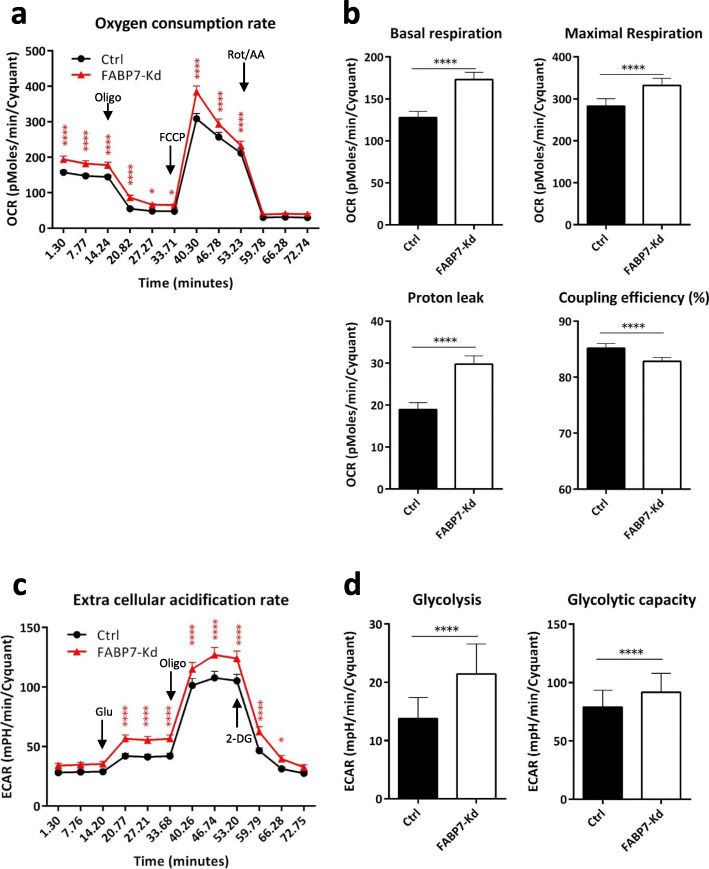
FABP7 knockdown (FABP7-Kd) increased oxygen consumption, proton leak, and glycolysis. **a** Oxygen consumption rate (OCR) curve of controls (Ctrl) and FABP7-Kd cells. Oligomycin (Oligo), FCCP, and rotenone/antimycin A (Rot/AA) were added sequentially. **b** Estimated mitochondrial basal respiration (upper left), maximal respiration (upper right), proton leak (lower left), and coupling efficacy (lower right) of Ctrl and FABP7-Kd. **c** Extracellular acidification rate (ECAR) curve of Ctrl and FABP7-Kd. Glucose (Glu), oligo, and 2-deoxy-glucose (2-DG) were added sequentially. **d** Estimated glycolysis and glycolytic capacity of Ctrl and FABP7-Kd. Error bars, SD; *p < 0.05, ***p < 0.001, ****p < 0.0001; *n* = 3. Each experiment consisted of 20 technical replicates for each condition

UCP1 function causes increased proton leak at the inner mitochondrial membrane, resulting in increased mitochondrial respiration rate and glycolysis to compensate energy expenditure [12, 13, 27, 28]. Flux analysis showed that FABP7 knockdown increased mitochondrial basal respiration and maximal respiration (Fig. 4a, b). Most importantly, FABP7 knockdown increased proton leak from mitochondria and reduced coupling efficacy with mitochondrial ETC without affecting ATP production (Fig. 4b). Glycolytic assay showed that FABP7 knockdown increased glycolysis, glycolytic capacity, and extracellular acidification (Fig. 4c, d). In contrast, FABP7 knockdown did not affect the fatty acid oxidation initiated either by the endogenous fatty acid storage or by the exogenously administrated palmitic acids (Fig. S3). Taken together, these results show that FABP7 knockdown results in similar metabolic profiles to beige fat with increased mitochondrial respiration and glycolysis to compensate the increased proton leak caused by UCP1.

### FABP7-knockdown-induced UCP1 depolarized mitochondrial membrane potential

**Fig. 5:**
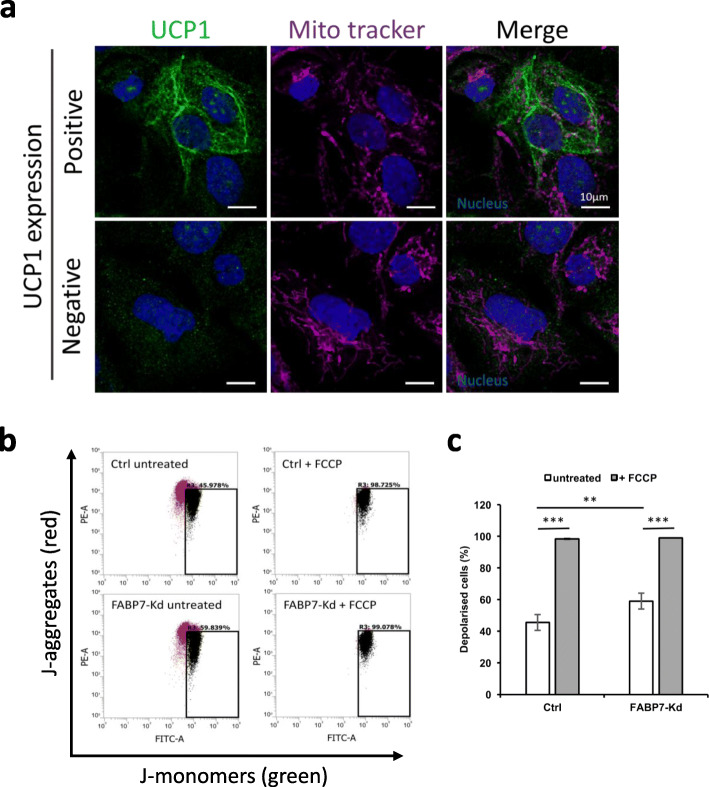
UCP1 caused focal depolarization of mitochondria. **a** Representative confocal microscopic images of UCP1-expressing cells (upper panels) and UCP1-negative cells (lower panels) acquired from FABP7 knockdowns (FABP7-Kd). UCP1 expression, polarized mitochondria (Mito Tracker), and nuclei are indicated in green, magenta, and blue, respectively. Scale bars; 10 μm. **b** Representative scatterplots of JC-1 assay. X and y axes show green (JC-1 monomer) and red (J-aggregate) fluorescence, respectively. The gate named depolarized was used for calculating the proportion of depolarized cells. **c** Proportion of depolarized cells in Ctrl and FABP7-Kd calculated through JC-1 assay. Error bars, SD; **p < 0.01, *n* = 6

To test further that the UCP1 was physiologically functional, we assessed the correlation of the spatial distribution of UCP1 with the focal depolarization of the mitochondrial membrane. For this purpose, we focused on the principle that Mito Tracker Red could preferentially label well-polarized mitochondria. In cells not expressing UCP1, polarized mitochondria (labeled with Mito Tracker Red) exhibited linear-like spatial distribution in the cytoplasm (Fig. 5a, lower panels). Conversely, in UCP1-expressing cells, polarized mitochondria exhibited fragmented distribution while UCP1 exhibited linear-like distribution (Fig. 5a, lower panels). Notably, UCP1 did not co-localize with the Mito Tracker positive-polarized part of mitochondria, and they exhibited a complementary distribution (Fig. 5a, upper panels). The co-staining using the higher concentration of Mito Tracker Red which was sufficient to visualize the whole structure of mitochondria ruled out the possibility of the ectopic UCP1 expression (Fig. S4).

This complementary pattern between UCP1 and polarized mitochondria suggested that UCP1 caused focal depolarization within the mitochondrial membrane. We then quantified the proportion of cells containing depolarized mitochondria using JC-1 mitochondrial dye. JC-1 trapped in mitochondria will lose red fluorescence in response to the depolarization of the mitochondrial membrane. The proportion of the depolarized cells which appeared in the segment of Green^high^/Red^low^ significantly increased among FABP7 knockdowns (Fig. 5), demonstrating that active dissipation of the ETC-generated proton gradient underlies focal depolarization of mitochondrial membrane when FABP7 function is disrupted.

### FABP7 knockdown sensitized cancer cells to hypoxia and γ-irradiation

**Fig. 6:**
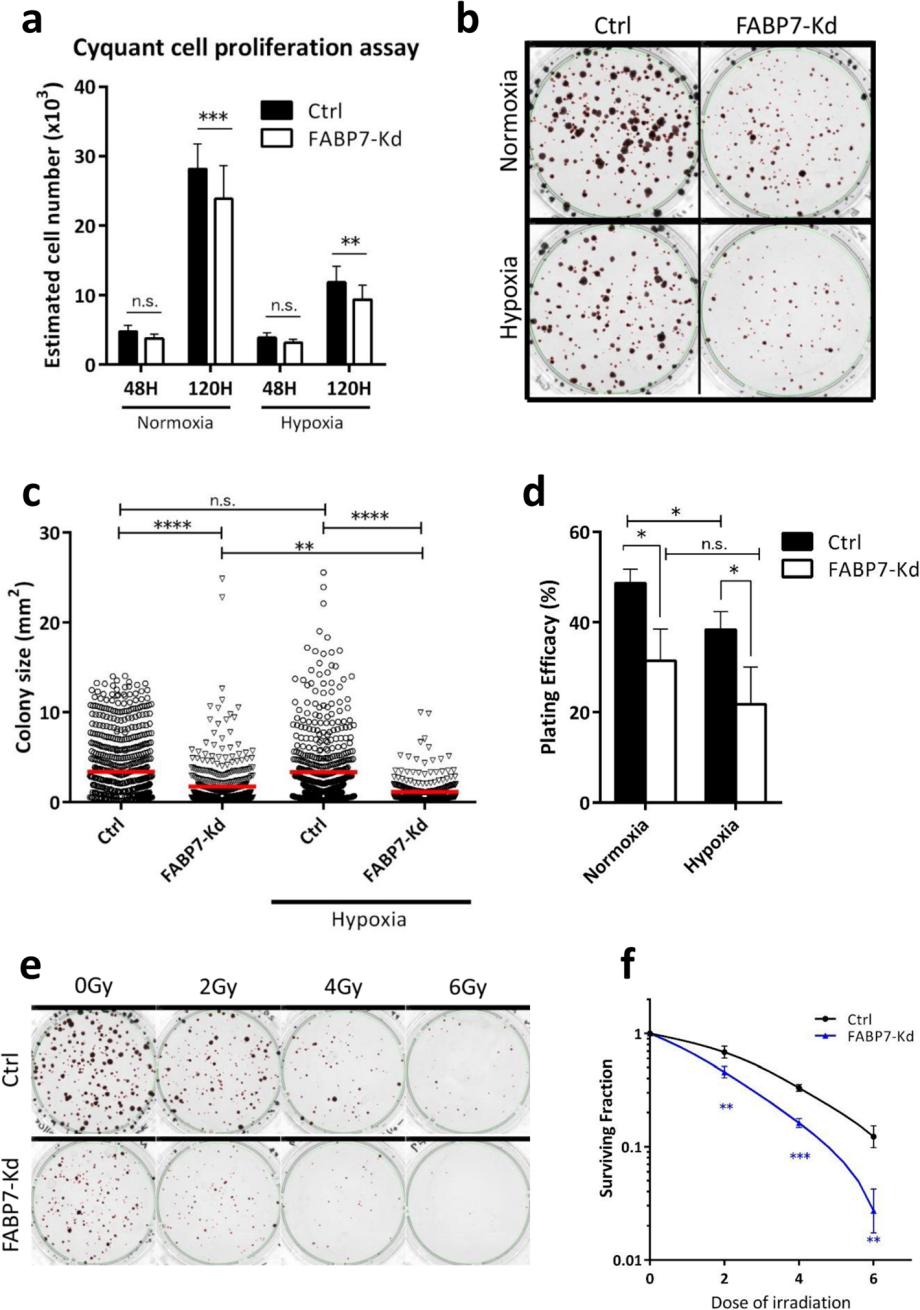
FABP7 knockdown (FABP7-Kd) inhibited cancer cell growth after hypoxia or ionizing radiation. **a** Comparison of cell proliferation between control (Ctrl) and FABP7-Kd under normoxia and hypoxia (0.1% O_2_). **b**–**d** Clonogenic assay performed under normoxia and under hypoxia-reoxygenation (72 h of 0.1% O_2_ followed by normoxia). **b** Representative image of colonies. **c** Distributions of colony size. Red bars show averaged colony size. **d** Plating efficacy of Ctrl and FABP7-Kd. **e**, **f** Clonogenic assay performed with/without ionizing radiation. **e** Representative image of colonies. **f** Calculated surviving fractions of Ctrl and FABP7-Kd. Error bars, SD; *p < 0.05, **p < 0.01, ***p < 0.001, ****p < 0.001; *n* = 3

The effect of the FABP7 knockdown on the viability of cancer cells was tested. FABP7 knockdown increased peroxidized lipid accumulation, with no further increase after hypoxia or γ-irradiation (Fig. S5a). In cell-cycle analysis, FABP7 knockdown increased the proportion of cells in sub-G1 phase (Fig. S5a, b) without affecting overall distribution of G0/G1, S, and G2/M phases (Fig. S5c). Since the increase in sub-G1 phase is known to reflect the accumulation of fragmented DNAs [29], it was likely that elevated lipid peroxidation increased DNA damage and cell death. Consistently, under both normoxia and hypoxia, FABP7 knockdown inhibited cell proliferation (Fig. 6a). Clonogenic assays showed more clearly that FABP7 knockdown significantly inhibited cell growth with a reduction in average colony size in normoxia and after exposure to hypoxia (0.1% O_2_, 72 h) (Fig. 6b, c). The knockdown of FABP7 also significantly reduced colony number under normoxia and hypoxia (Fig. 6d). Because FABP7-knockdown cells exhibited increased lipid peroxidation, we tested their sensitivity to γ-irradiation. At all tested γ-irradiation doses (2 Gy, 4 Gy, and 6 Gy), FABP7-knockdown cells had lower colony forming ability (Fig. 6e, f).

In contrast to these in vitro experimental results, we found no substantial correlation of FABP7 and UCP1 expression on patient survival in the METABRIC and TCGA breast cancer cohorts. Univariate and multivariate analysis using the METABRIC and TCGA breast cancer cohorts identified only hypoxia score as a significant prognostic factor (Table 1). UCP1 did not have a significant impact on patient prognosis (Table 1). Higher FABP7 expression was associated with better progression-free survival in ER-positive patients in TCGA cohort, but this result was not reproduced in METABRIC cohort (Table 1 and S1). Survival curves confirmed that UCP1 expression in breast tumors was not associated with overall survival either in ER-positive or ER-negative patients (Fig. S6).

**Table 1:** Prognostic impact of hypoxia score, UCP1, and FABP7

	Univariate		Multivariate	
	HR	*p*-value	HR	*p*-value
**METABRIC**				
All cases (*N* = 1904)				
Hypoxia-score	1.30 (1.10-1.50)	<0.001***	1.43 (1.21-1.68)	<0.001***
UCP1	0.94 (0.84-1.10)	0.27	0.96 (0.86-1.08)	0.515
FABP7	0.99 (0.94-1.00)	0.504	0.95 (0.90-0.99)	0.02*
ER+ (*N* = 1445)				
Hypoxia-score	1.40 (1.20-1.80)	<0.001***	1.47 (1.18-1.80)	<0.001***
UCP1	0.99 (0.88-1.10)	0.908	1.01 (0.90-1.10)	0.875
FABP7	0.97 (0.89-1.10)	0.558	0.94 (0.85-1.00)	0.214
ER- (*N* = 429)				
Hypoxia-score	1.20 (0.88-1.60)	0.246	1.24 (0.89-1.70)	0.201
UCP1	0.62 (0.28-1.40)	0.241	0.64 (0.29-1.40)	0.278
FABP7	0.95 (0.90-1.00)	0.114	0.94 (0.88-1.00)	0.053
**TCGA**				
All cases (*N* = 960)				
hypoxia-score	1.50 (1.00-2.10)	0.028*	2.19 (1.46-3.28)	<0.001***
UCP1	1.00 (0.92-1.10)	0.763	1.04 (0.95-1.14)	0.403
FABP7	0.92 (0.87-0.97)	0.003**	0.89 (0.84-0.94)	<0.001***
ER+ (*N* = 708)				
hypoxia-score	1.40 (0.80-2.40)	0.246	1.50 (0.80-2.64)	0.216
UCP1	1.00 (0.94-1.20)	0.403	1.10 (0.96-1.16)	0.244
FABP7	0.80 (0.71-0.89)	<0.001***	0.80 (0.72-0.89)	<0.001***
ER- (*N* = 208)				
hypoxia-score	1.20 (0.57-2.6)	0.601	1.59 (0.71-3.50)	0.255
UCP1	1.00 (0.58-1.80)	0.944	1.08 (0.60-1.90)	0.799
FABP7	0.93 (0.86-1.00)	0.092	0.92 (0.84-1.00)	0.053

*p < 0.05, **p < 0.01, and ***p < 0.001

### UCP1 increased cellular temperature

**Fig. 7:**
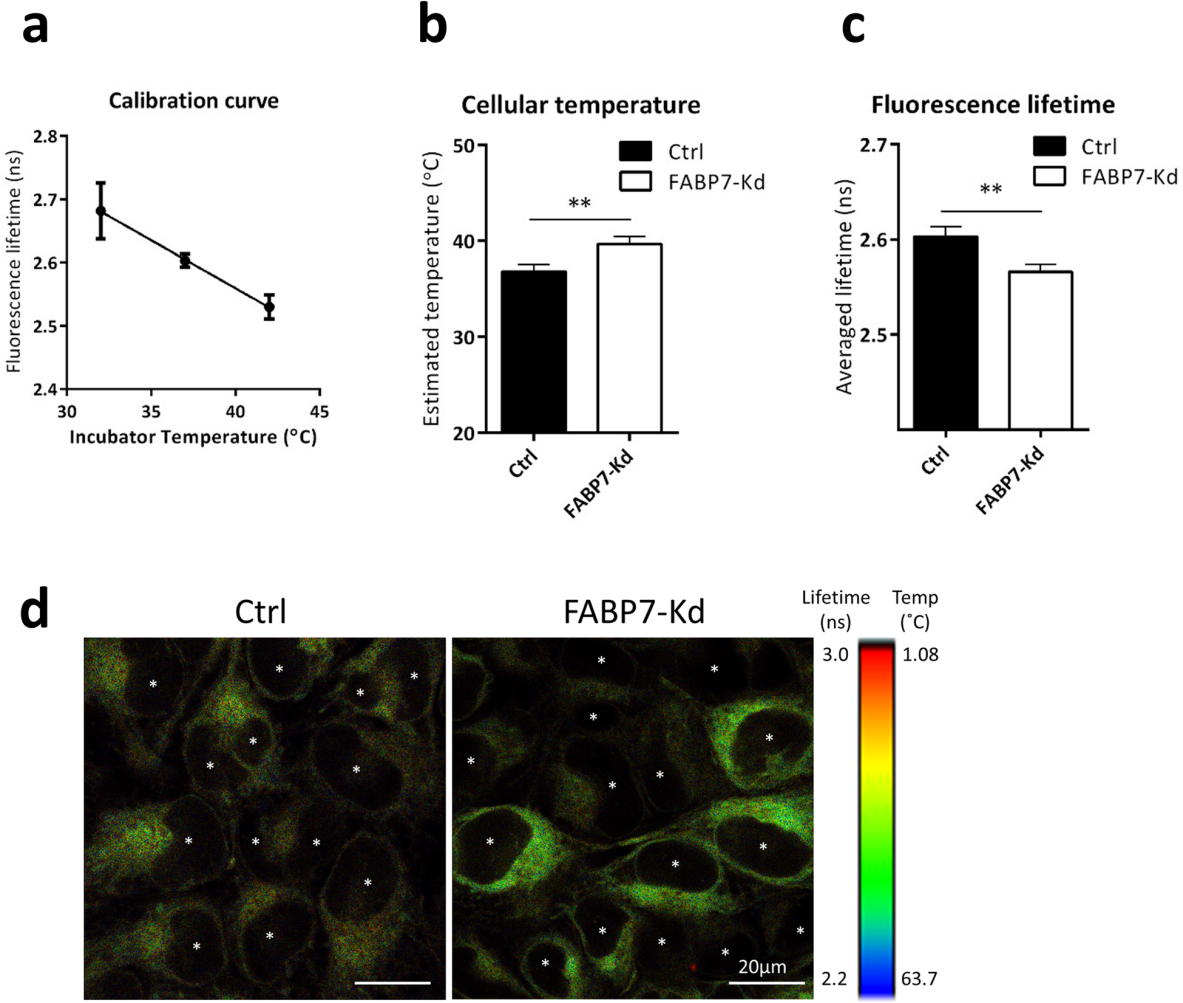
FABP7 knockdown (FABP7-Kd) increased cellular temperature. **a** Calibration curve of T probe generated using control cells (Ctrl). X and y axes show incubator temperatures and average fluorescence lifetime, respectively. **b** Average fluorescence lifetimes of Ctrl and FABP7-Kd. **c** Calculated cellular temperature of Ctrl (37 °C) and FABP7-Kd. **d** Representative images of fluorescence lifetime imaging microscopy. Color scale indicates estimated temperature. Scale bars; 20 μm. Asterisks indicate nuclei locations. Error bars, SD; **p < 0.01, *n* = 3

Finally, we measured the difference of cellular temperature by using the thermosensitve probe (T probe) that shortens its fluorescence lifetime as cellular temperature increased [5]. We observed the expected inverse linear correlation between the fluorescent lifetime of our T probe and control-cell temperatures (Fig. 7a), indicating that the probe is appropriate for use in our experiment.

The shorter fluorescence lifetime of FABP7-knockdown cells indicated a higher temperature compared with control cells (37 °C) (Fig. 7b). We estimated the temperature in FABP7-knockdown cells to be 39.5 °C (difference from control was 2.47 ± 0.42 °C, Fig. 7c). Elevated temperature in FABP7-knockdown cells was also confirmed using fluorescence life-time imaging (indicated as brighter color in Fig. 7d). Furthermore, the distribution of warm cells was coincided with the pattern of UCP1-expressing cells (Fig. 7d). Thus, FABP7-knockdown-induced UCP1 activated autonomous heat production in breast cancer cells.

## Discussion

Here, we described a new metabolic feature in breast cancer, namely, that blocking hypoxia-inducible FABP7 triggers UCP1-mediated thermogenesis. We confirmed the existence of autonomous thermogenesis in breast cancer cells using a novel thermosensitive fluorescent probe.

In beige adipocytes, transcriptional regulator PRDM16 and co-factor PGC1α regulate differentiation and contribute to basal UCP1 expression [30]. In addition, increasing cAMP levels maximizes UCP1 induction [10]. We observed that FABP7 knockdown led to the upregulation of PRDM16 and PGC1α under normoxia and elevated pCREB levels under hypoxia. FABP7 functions not only as a fatty acid transporter but also as a regulator of differentiation in brain tissue. Thus, an effect of FABP7 on differentiation in the cancer cells is plausible. Oxidized fatty acids and their derivatives epigenetically induced beige fat differentiation through DNA or histone modification [31]. Similarly, oxidized fatty acids and derivatives directly interact with transcriptional factors that upregulate UCP1 transcription, such as peroxisome proliferator-activated receptor [32–35]. Hence, our findings suggested that the increased fatty acid peroxidation upon FABP7 knockdown might contribute to the induction of UCP1, although the precise mechanism should be further investigated. Given that FABP7 is a fatty acid transporter, identifying the fatty acid derivatives which could directly regulate UCP1-mediated thermogenesis would be also important.

Generally, UCP1 is considered to be expressed exclusively in adult brown or beige adipocytes. Therefore, it is widely used as a specific marker for brown and beige adipocytes. Although several studies have reported ectopic UCP1 expression in multiple cancers, including breast cancer [36–41], the functionality of UCP1 has never been elucidated. In the present study, we observed the depolarization of mitochondrial membrane potential, a phenomenon associated with the uncoupling by UCP1. In addition, we observed the increase in glucose catabolism, mitochondrial respiration, and mitochondrial proton leak that were key metabolic features of beige adipocytes. Furthermore, we confirmed that the anti-UCP1 antibody we used specifically recognized UCP1 protein by using a blocking peptide although the antibody is well accepted in detecting human UCP1 [42]. Most importantly, we observed a rise in cellular temperature upon UCP1 induction. An earlier study explained that the higher temperature of breast tumors was due to increased blood flow compared to normal tissue [3]. Our study demonstrated that heat generated by cancer cells could also contribute to an increase in tissue temperature, although it would be difficult to deconvolute the relative effects in vivo.

Beside the role in thermogenesis, UCP1 has been generally considered to protect the cells from ROS injury by diminishing the proton flux of mitochondrial complex V [11]. However, FABP7-knockdown cells exhibited slower growth and became more vulnerable to oxidative stresses (e.g., hypoxia-reoxygenation and ionizing radiation) despite the putative protective role of UCP1 against ROS damage. This suggested that FABP7 might be more effective in protecting cancer cells from ROS damage than UCP1. Consistently, strong UCP1 expression was observed in both normal mammary glands and well-differentiated tumors, whereas strong FABP7 expression was observed in poorly differentiated and severely hypoxic tumors. There is also a striking correlation of expression of each gene with specific types of breast cancer, ER positive to UCP1, and ER negative to FABP7. This confounds the associations with outcome, as there are well-reported differences in outcome of these types of breast cancer [43–45]. In the survival analysis using METABRIC and TCGA cohort, there was no difference in outcome depending on the expression of UCP1 and FABP7. Although it should be noted that the number of either FABP7 high or UCP1 high cases we defined was so small, these findings suggest that upregulating FABP7 may be protective for the more aggressive subtype (Fig. S7), whereas UCP1 is associated with a different mitochondrial metabolism in slower growing tumors. In a study using a mouse xenograft model, UCP1 expression was essential for tumorigenic ability, and its expression decreased in accordance with tumor progression [40]. Since there are greater demands for ATP in rapidly proliferating poorly differentiated tumors, there would be a disadvantage in uncoupling between ETC and mitochondrial complex V.

From the immunological point of view, heat has been well recognized as a major modifier of systemic immune responses [16]. For example, a recent study showed that a thermal sensory pathway, HSP90-α4-integrin axis, promoted T lymphocyte trafficking and enhanced immune surveillance during infection [17]. Therefore, the effect of heat on tumor immune responses potentially links thermogenesis and the outcome of immune therapy including immune checkpoint inhibition (ICI). Intriguingly, we recently found that the expression of FABP7 and other genes related to PUFA transport was associated with the enrichment of molecular pathways related to ICI in breast cancer tissues [46]. Therefore, it is of interest to investigate whether UCP1-mediated thermogenesis could enhance the benefit of ICI by functioning as “endogenous hyperthermia.”

Curiously, UCP1 was only expressed in approximately 20–30% of cancer cells even at the highest induction rates. Beige adipocytes could also appear in patchy pattern within white adipose tissue upon the stimulation such as cold-induced sympathetic nerve activation. The reason that they do not completely replace white adipocytes is not well understood. The cancer cells seemed to mimic this typical distribution pattern of beige adipocytes, reflecting the complexity of metabolic adaptation in cancer cells. FABP7 is also known to exhibit heterogenous expression in brain tissue: its expression is limited to neural stem cells or progenitor cells [47]. Therefore, we could posit that there are subsets of cells undergoing metabolic adaptation through different quantitative/qualitative regulation of transcription. The more precise mechanism underlying this phenomenon could be addressed by single cell-based assays in future experiments. This raises the possibility that there could be a co-operative effect on tumor growth by having different metabolic populations.

## Conclusions

We observed that FABP7 knockdown induced UCP1-mediated thermogenesis in a breast cancer cell line. FABP7 knockdown increased the susceptibility to hypoxia and γ-irradiation, providing a potential therapeutic window for breast cancer. Since heat can also affect tumor immunity, it would be of interest to examine how the thermogenesis by cancer cells affects the response to immune therapy. This is particularly the case as ER negative tumors which tend to express high FABP7 are the ones in which anti-PD1 therapy seems to be more effective. Although more studies are needed to elucidate the molecular mechanisms linking FABP7-related fatty acid metabolism and UCP1 induction, our findings illustrate a new metabolic adaption of cancer cells that involves heat production similar to that used by beige adipocytes. Taken together, FABP7 could be a potential target for cancer therapy that affects the sensitivity to oxidative stress and γ-irradiation although its prognostic impact remains to be further investigated.

## Supplementary information

**Additional file 1: Figure S1**. **a** Association of FABP7, UCP1, and hypoxia ssGSEA quartile in the Metabric (n = 1904, upper panels) and TCGA (n = 960, lower panels) breast cancer cohorts. The scatter plots of all samples (left), ER+ tumors (middle) and ER negative tumors (right) are shown. X and y axes show UCP1 and FABP7 mRNA expression, respectively. Hypoxia ssGSEA quartiles were indicated using different colors (orange, green, blue and purple). **b** Correlation analyses of hypoxia ssGSEA with FABP7 (upper panels) and UCP1 expression (lower panels).

**Additional file 2: Figure S2**. Blocking peptide confirmed specificity of the anti-UCP1 antibody. a Western blot using the anti-UCP1 antibody with (left) and without addition of UCP1 peptide (right). b Immunofluorescence of FABP7 knockdown cells with (left) and without addition of UCP1 peptide (right). UCP1 and nuclei were stained with green and blue, respectively. Scale bars; 20 μm.

**Additional file 3: Figure S3**. Exogenous fatty acid oxidation (FAO) and endogenous FAO estimated by Seahorse XFe96. Left: Exogenous FAO is estimated as the difference between the oxygen consumption rate (OCR) with and without palmitate supplementation [FAO induced by exogenously supplied palmitate]. Right: endogenous FAO was estimated as the difference between the OCR with and without etmoxir (specific inhibitor of mitochondrial CPT-1) supplementation [FAO induced by endogenously supplied FAs].The growth media was replaced to the substrate-limited media (DMEM without sodium pyruvate supplemented with 0.5mM glucose, 1mM glutamine, 0.5mM L-carnitine and 1%FBS (pH 7.4 at 37 ˚C) 16hr prior to the assay. The substrate-limited media was replaced to FAO assay media: KHB (111mM NaCl, 4.7mM KCl, 1.25mM CaCl2, 2mM MgSO4, 1.2mM NaH2PO4) supplemented with 2.5mM glucose, 0.5 mM carnitine, and 5 mM HEPES and the cells were transferred to non-CO2 incubator (37 ˚C) 45 min prior to the assay. 40μM etomoxir was added 15 min prior to the assay and XF Palmitate-BSA FAO substrate or BSA were added just prior to the assay.

**Additional file 4: Figure S4**. Immunofluorescent image of UCP1 positive cells. To increase the sensitivity of Mito tracker, mitochondoria were stained with higher concentration of Mito tracker. Co-localization of UCP1 (green) and Mito tracker (magenta) was recognized as white signals (indicated by white arrows).

**Additional file 5: Figure S5**. FABP7-knockdown (FABP7-Kd) induced lipid peroxidation and led to the increase of sub-G1 phase in cell-cycle analysis. a Comparison of lipid peroxidation levels between control (Ctrl) and FABP7-Kd under normoxia, hypoxia (0.1% O2, 24 hr), and 24 hr after ionizing radiation (4Gy). b, c, d Cell-cycle analysis of Ctrl and FABP7-Kd. b Representative cell-cycle distribution. c Difference of the proportion of sub-G1 population. d Cell-cycle distribution without sub-G1 phase. Error bars, SD; *p < 0.05, **p < 0.01; n = 3.

**Additional file 6: Figure S6**. **a** Association of UCP1 mRNA expression in tumors with overall survival assessed through the METABRIC breast cancer cohort. Kaplan meier estimates using all cases (left), ER-positive cases (middle), ER-negative (right) were shown. UCP1-high and low were defined by k-means clustering (k=2). **b** the same analyses through the TCGA breast cancer cohort.

**Additional file 7: Figure S7**. Working hypothesis generated from this study.

**Additional file 8: Table S1**. Prognostic impact of hypoxia ssGSEA, UCP1 and FABP7

## Data Availability

All data are available from the corresponding author upon reasonable request.

## Abbreviations

UCP1: Uncoupling protein 1
FABP7: Fatty acid binding protein 7
ETC: Electron transport chain
ER: Estrogen receptor
PRDM16: PR/SET domain 16
PGC1α: Peroxisome proliferator-activated receptor gamma coactivator 1-alpha
cAMP: Cyclic AMP
PUFA: Polyunsaturated fatty acid
CREB: cAMP responsive element binding protein
T probe: Thermosensitve probe
FBS: Fetal bovine serum
FLIM: Fluorescence lifetime imaging microscopy
METABRIC: Molecular Taxonomy of Breast Cancer International Consortium
TCGA: The Cancer Genome Atlas
ssGSEA: Single-sample Gene Set Enrichment Analysis
KM: Kaplan-Meier
