# Correction to: Disruption of hypoxia-inducible fatty acid binding protein 7 induces beige fat-like differentiation and thermogenesis in breast cancer cells

**DOI:** 10.1186/s40170-020-00224-7

**Published:** 2020-08-10

**Authors:** Masahiro Kawashima, Karim Bensaad, Christos E. Zois, Alessandro Barberis, Esther Bridges, Simon Wigfield, Christoffer Lagerholm, Ruslan I. Dmitriev, Mariko Tokiwa, Masakazu Toi, Dmitri B. Papkovsky, Francesca M. Buffa, Adrian L. Harris

**Affiliations:** 1 Department of Oncology, Molecular Oncology Laboratories, Weatherall Institute of Molecular Medicine, University of Oxford, John Radcliffe Hospital, Oxford, OX3 9DS UK; 2 Department of Oncology, Computational Biology and Integrative Genomics Lab, CRUK/MRC Institute for Radiation Oncology, University of Oxford, Old Road Campus Research Building, Roosvelt Drive, Oxford, OX3 7DQ UK; 3 Wolfson Imaging Centre, Weatherall Institute of Molecular Medicine, University of Oxford, John Radcliffe Hospital, Oxford, OX3 9DS UK; 4 School of Biochemistry and Cell Biology, University College Cork, Cavanagh Pharmacy Building, 1.28, College Road, Cork, Ireland; 5 Department of Breast Surgery, Graduate School of Medicine, Kyoto University, 54 Shogoin-Kawahara-cho, Sakyo-ku, Kyoto, 606 8507 Japan; 6 Institute for Regenerative Medicine, I.M. Sechenov First Moscow State University, Moscow, Russian Federation

**Correction to: Cancer Metab 8, 13 (2020)**

**https://doi.org/10.1186/s40170-020-00219-4**

Following publication of the original article [1], the authors identified an error in Fig. 7. The correct figure is given below.

**Fig. 7:**
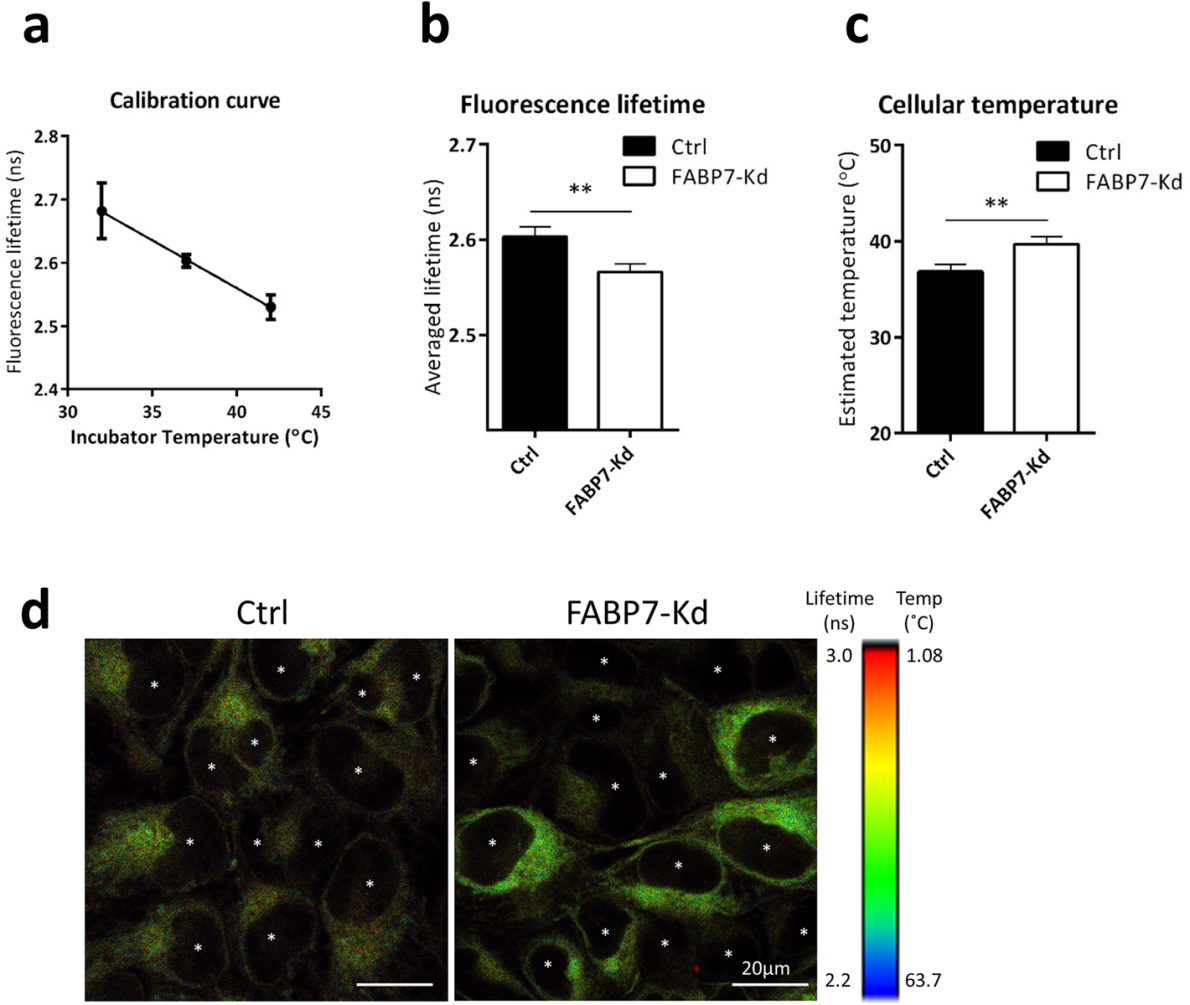
FABP7 knockdown (FABP7-Kd) increased cellular temperature. a Calibration curve of T probe generated using control cells (Ctrl). X and y axes show incubator temperatures and average fluorescence lifetime, respectively. b Average fluorescence lifetimes of Ctrl and FABP7-Kd. c Calculated cellular temperature of Ctrl (37 °C) and FABP7-Kd. d Representative images of fluorescence lifetime imaging microscopy. Color scale indicates estimated temperature. Scale bars; 20 μm. Asterisks indicate nuclei locations. Error bars, SD; **p < 0.01, *n* = 3
