# Supplementary material for: Disruption of hypoxia-inducible fatty acid binding protein 7 induces beige fat-like differentiation and thermogenesis in breast cancer cells
Source: Cancer Metab. 2020 Jul 6;8:13. doi: 10.1186/s40170-020-00219-4 (PMC7336487; doi:10.1186/s40170-020-00219-4)
Supplement: Supplementary file 1 — Additional file 1: Figure S1. a Association of FABP7, UCP1, and hypoxia ssGSEA quartile in the Metabric (n = 1904, upper panels) and TCGA (n = 960, lower panels) breast cancer cohorts. The scatter plots of all samples (left), ER+ tumors (middle) and ER negative tumors (right) are shown. X and y axes show UCP1 and FABP7 mRNA expression, respectively. Hypoxia ssGSEA quartiles were indicated using different colors (orange, green, blue and purple). b Correlation analyses of hypoxia ssGSEA with FABP7 (upper panels) and UCP1 expression (lower panels). [file 40170_2020_219_MOESM1_ESM.pptx]

## Slide 1
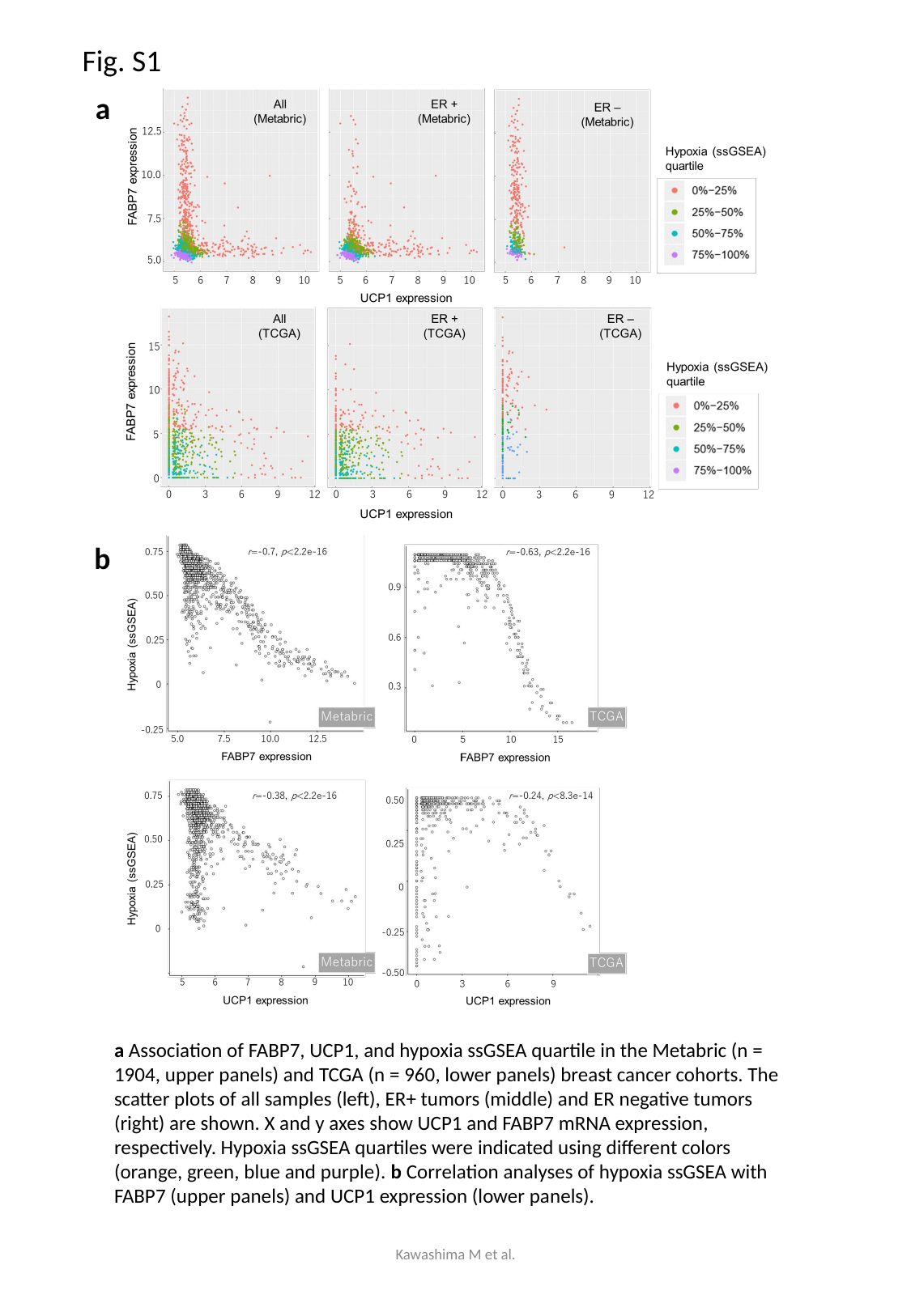

Fig. S1
a
b
a Association of FABP7, UCP1, and hypoxia ssGSEA quartile in the Metabric (n = 1904, upper panels) and TCGA (n = 960, lower panels) breast cancer cohorts. The scatter plots of all samples (left), ER+ tumors (middle) and ER negative tumors (right) are shown. X and y axes show UCP1 and FABP7 mRNA expression, respectively. Hypoxia ssGSEA quartiles were indicated using different colors (orange, green, blue and purple). b Correlation analyses of hypoxia ssGSEA with FABP7 (upper panels) and UCP1 expression (lower panels).
Kawashima M et al.
