# Supplementary material for: Disruption of hypoxia-inducible fatty acid binding protein 7 induces beige fat-like differentiation and thermogenesis in breast cancer cells
Source: Cancer Metab. 2020 Jul 6;8:13. doi: 10.1186/s40170-020-00219-4 (PMC7336487; doi:10.1186/s40170-020-00219-4)
Supplement: Supplementary file 2 — Additional file 2: Figure S2. Blocking peptide confirmed specificity of the anti-UCP1 antibody. a Western blot using the anti-UCP1 antibody with (left) and without addition of UCP1 peptide (right). b Immunofluorescence of FABP7 knockdown cells with (left) and without addition of UCP1 peptide (right). UCP1 and nuclei were stained with green and blue, respectively. Scale bars; 20 μm. [file 40170_2020_219_MOESM2_ESM.pptx]

## Slide 1
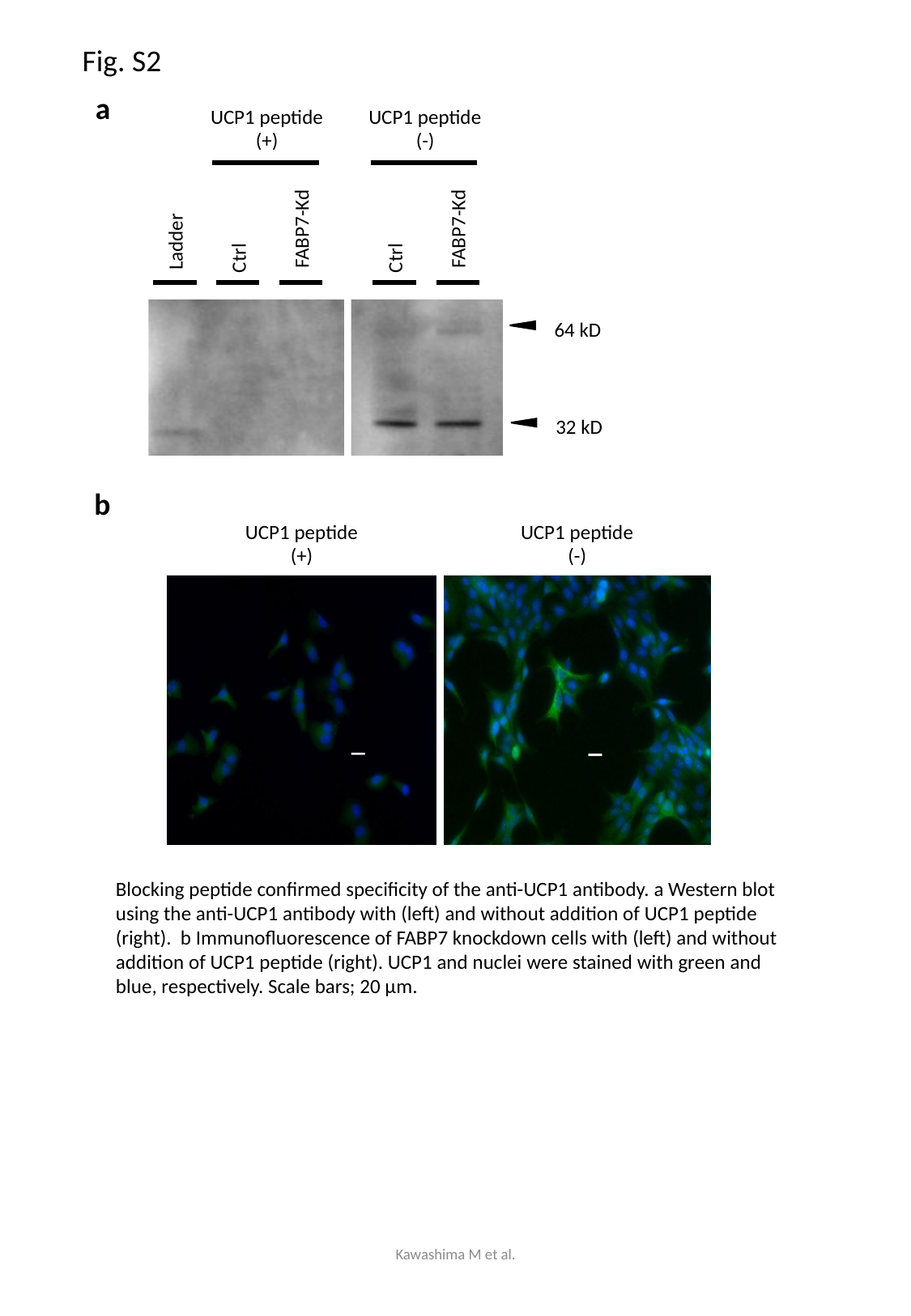

Fig. S2
a
UCP1 peptide
(+)
UCP1 peptide
(-)
FABP7-Kd
FABP7-Kd
Ladder
Ctrl
Ctrl
64 kD
32 kD
b
UCP1 peptide
(+)
UCP1 peptide
(-)
Blocking peptide confirmed specificity of the anti-UCP1 antibody. a Western blot using the anti-UCP1 antibody with (left) and without addition of UCP1 peptide (right). b Immunofluorescence of FABP7 knockdown cells with (left) and without addition of UCP1 peptide (right). UCP1 and nuclei were stained with green and blue, respectively. Scale bars; 20 μm.
Kawashima M et al.
