# Supplementary material for: Disruption of hypoxia-inducible fatty acid binding protein 7 induces beige fat-like differentiation and thermogenesis in breast cancer cells
Source: Cancer Metab. 2020 Jul 6;8:13. doi: 10.1186/s40170-020-00219-4 (PMC7336487; doi:10.1186/s40170-020-00219-4)
Supplement: Supplementary file 3 — Additional file 3: Figure S3. Exogenous fatty acid oxidation (FAO) and endogenous FAO estimated by Seahorse XFe96. Left: Exogenous FAO is estimated as the difference between the oxygen consumption rate (OCR) with and without palmitate supplementation [FAO induced by exogenously supplied palmitate]. Right: endogenous FAO was estimated as the difference between the OCR with and without etmoxir (specific inhibitor of mitochondrial CPT-1) supplementation [FAO induced by endogenously supplied FAs].The growth media was replaced to the substrate-limited media (DMEM without sodium pyruvate supplemented with 0.5mM glucose, 1mM glutamine, 0.5mM L-carnitine and 1%FBS (pH 7.4 at 37 ˚C) 16hr prior to the assay. The substrate-limited media was replaced to FAO assay media: KHB (111mM NaCl, 4.7mM KCl, 1.25mM CaCl2, 2mM MgSO4, 1.2mM NaH2PO4) supplemented with 2.5mM glucose, 0.5 mM carnitine, and 5 mM HEPES and the cells were transferred to non-CO2 incubator (37 ˚C) 45 min prior to the assay. 40μM etomoxir was added 15 min prior to the assay and XF Palmitate-BSA FAO substrate or BSA were added just prior to the assay. [file 40170_2020_219_MOESM3_ESM.pptx]

## Slide 1
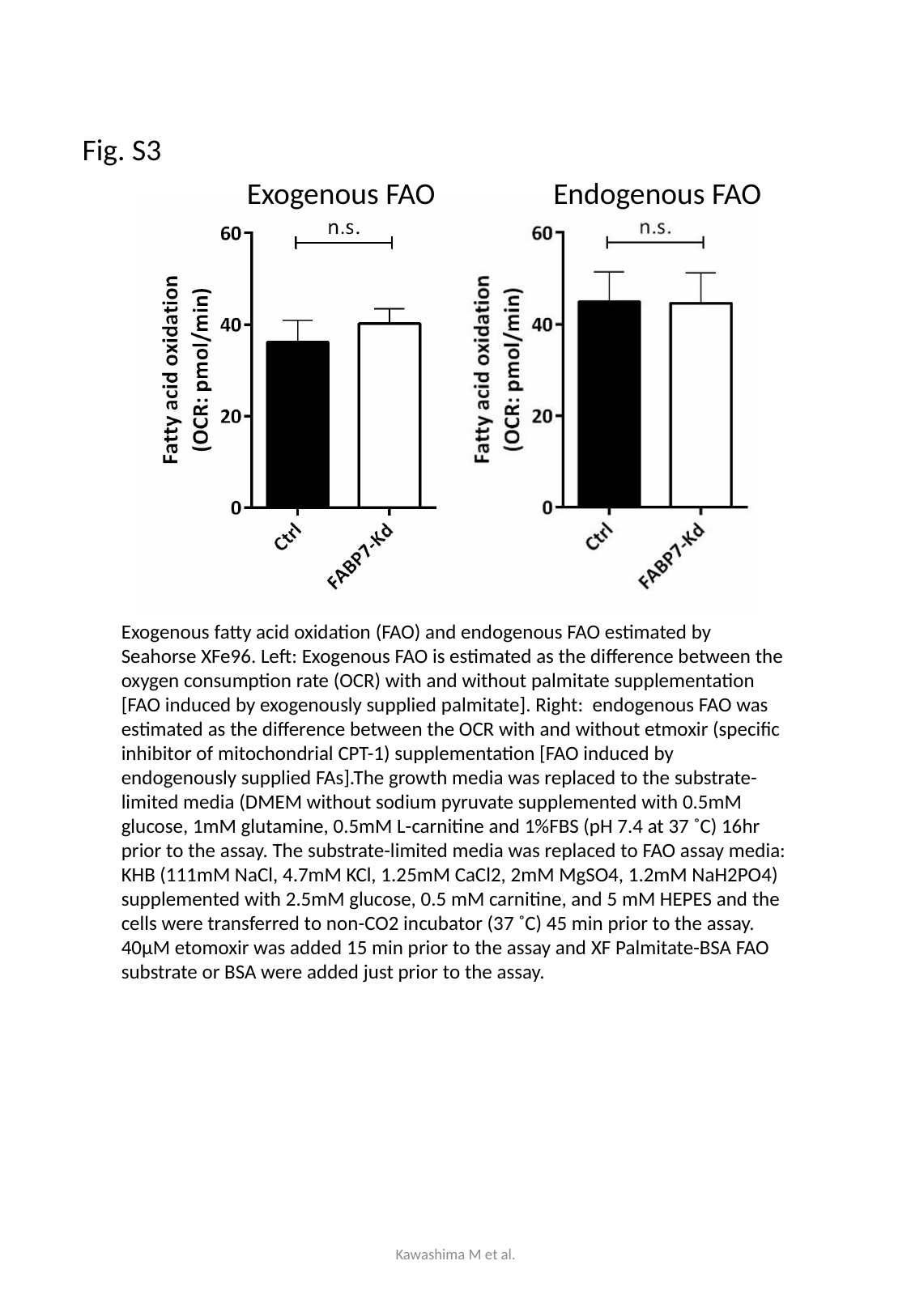

Fig. S3
Exogenous FAO
Endogenous FAO
Exogenous fatty acid oxidation (FAO) and endogenous FAO estimated by Seahorse XFe96. Left: Exogenous FAO is estimated as the difference between the oxygen consumption rate (OCR) with and without palmitate supplementation [FAO induced by exogenously supplied palmitate]. Right: endogenous FAO was estimated as the difference between the OCR with and without etmoxir (specific inhibitor of mitochondrial CPT-1) supplementation [FAO induced by endogenously supplied FAs].The growth media was replaced to the substrate-limited media (DMEM without sodium pyruvate supplemented with 0.5mM glucose, 1mM glutamine, 0.5mM L-carnitine and 1%FBS (pH 7.4 at 37 ˚C) 16hr prior to the assay. The substrate-limited media was replaced to FAO assay media: KHB (111mM NaCl, 4.7mM KCl, 1.25mM CaCl2, 2mM MgSO4, 1.2mM NaH2PO4) supplemented with 2.5mM glucose, 0.5 mM carnitine, and 5 mM HEPES and the cells were transferred to non-CO2 incubator (37 ˚C) 45 min prior to the assay. 40μM etomoxir was added 15 min prior to the assay and XF Palmitate-BSA FAO substrate or BSA were added just prior to the assay.
Kawashima M et al.
