# Supplementary material for: Disruption of hypoxia-inducible fatty acid binding protein 7 induces beige fat-like differentiation and thermogenesis in breast cancer cells
Source: Cancer Metab. 2020 Jul 6;8:13. doi: 10.1186/s40170-020-00219-4 (PMC7336487; doi:10.1186/s40170-020-00219-4)
Supplement: Supplementary file 4 — Additional file 4: Figure S4. Immunofluorescent image of UCP1 positive cells. To increase the sensitivity of Mito tracker, mitochondoria were stained with higher concentration of Mito tracker. Co-localization of UCP1 (green) and Mito tracker (magenta) was recognized as white signals (indicated by white arrows). [file 40170_2020_219_MOESM4_ESM.pptx]

## Slide 1
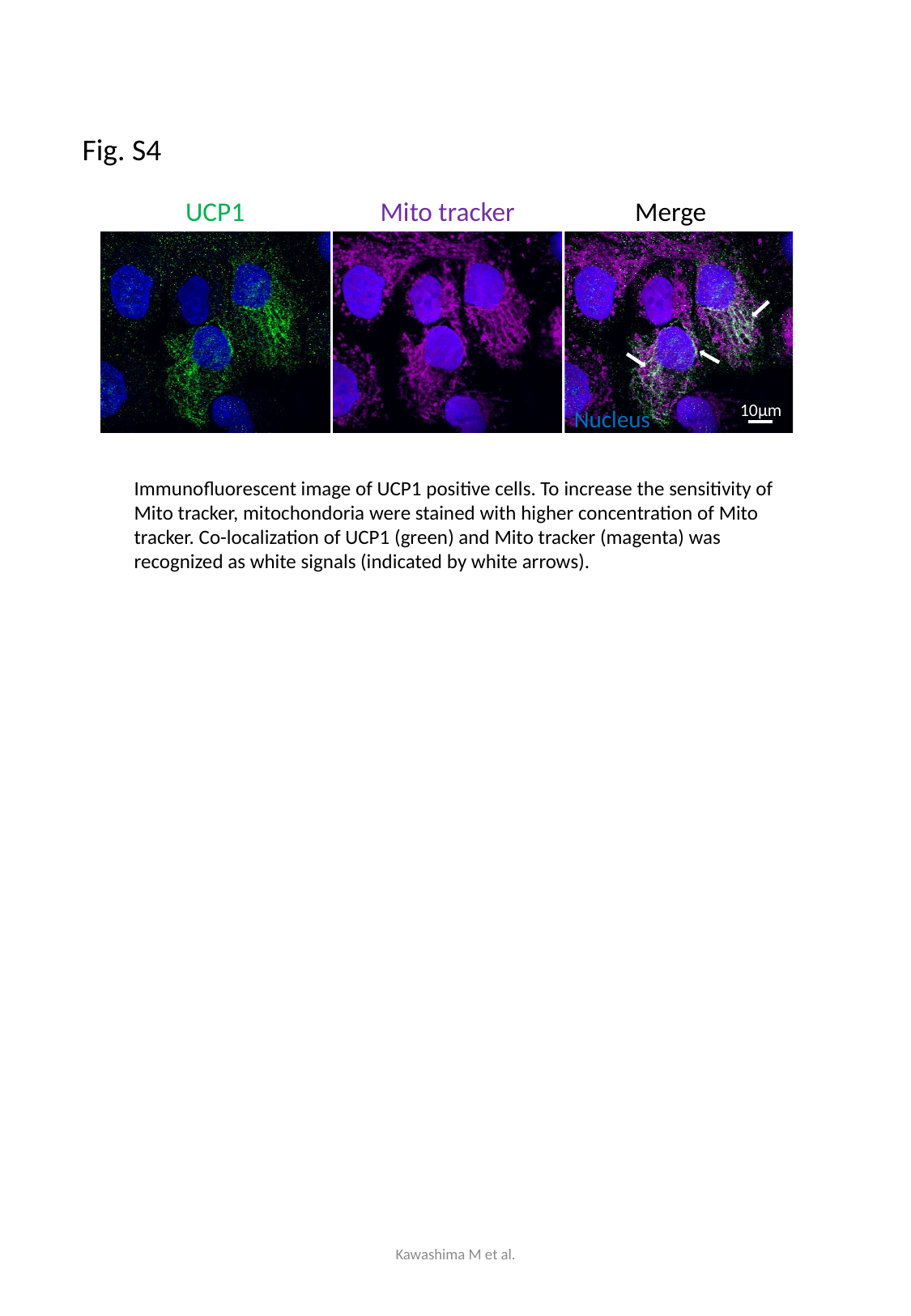

Fig. S4
Mito tracker
Merge
UCP1
10μm
Nucleus
Immunofluorescent image of UCP1 positive cells. To increase the sensitivity of Mito tracker, mitochondoria were stained with higher concentration of Mito tracker. Co-localization of UCP1 (green) and Mito tracker (magenta) was recognized as white signals (indicated by white arrows).
Kawashima M et al.
