# Supplementary material for: Disruption of hypoxia-inducible fatty acid binding protein 7 induces beige fat-like differentiation and thermogenesis in breast cancer cells
Source: Cancer Metab. 2020 Jul 6;8:13. doi: 10.1186/s40170-020-00219-4 (PMC7336487; doi:10.1186/s40170-020-00219-4)
Supplement: Supplementary file 5 — Additional file 5: Figure S5. FABP7-knockdown (FABP7-Kd) induced lipid peroxidation and led to the increase of sub-G1 phase in cell-cycle analysis. a Comparison of lipid peroxidation levels between control (Ctrl) and FABP7-Kd under normoxia, hypoxia (0.1% O2, 24 hr), and 24 hr after ionizing radiation (4Gy). b, c, d Cell-cycle analysis of Ctrl and FABP7-Kd. b Representative cell-cycle distribution. c Difference of the proportion of sub-G1 population. d Cell-cycle distribution without sub-G1 phase. Error bars, SD; *p < 0.05, **p < 0.01; n = 3. [file 40170_2020_219_MOESM5_ESM.pptx]

## Slide 1
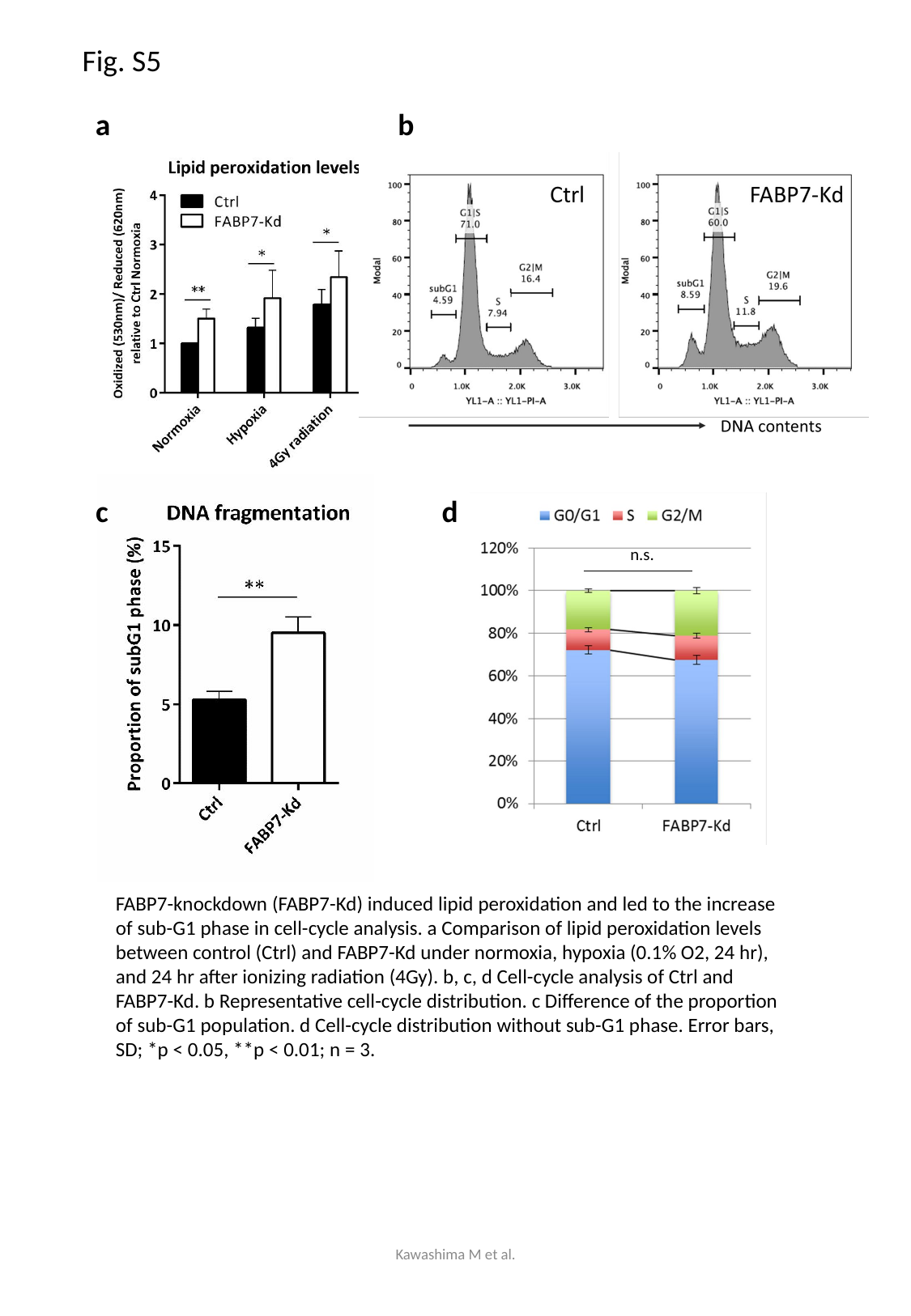

Fig. S5
a
b
c
d
n.s.
FABP7-knockdown (FABP7-Kd) induced lipid peroxidation and led to the increase of sub-G1 phase in cell-cycle analysis. a Comparison of lipid peroxidation levels between control (Ctrl) and FABP7-Kd under normoxia, hypoxia (0.1% O2, 24 hr), and 24 hr after ionizing radiation (4Gy). b, c, d Cell-cycle analysis of Ctrl and FABP7-Kd. b Representative cell-cycle distribution. c Difference of the proportion of sub-G1 population. d Cell-cycle distribution without sub-G1 phase. Error bars, SD; *p < 0.05, **p < 0.01; n = 3.
Kawashima M et al.
