# Supplementary material for: Disruption of hypoxia-inducible fatty acid binding protein 7 induces beige fat-like differentiation and thermogenesis in breast cancer cells
Source: Cancer Metab. 2020 Jul 6;8:13. doi: 10.1186/s40170-020-00219-4 (PMC7336487; doi:10.1186/s40170-020-00219-4)
Supplement: Supplementary file 6 — Additional file 6: Figure S6. a Association of UCP1 mRNA expression in tumors with overall survival assessed through the METABRIC breast cancer cohort. Kaplan meier estimates using all cases (left), ER-positive cases (middle), ER-negative (right) were shown. UCP1-high and low were defined by k-means clustering (k=2). b the same analyses through the TCGA breast cancer cohort. [file 40170_2020_219_MOESM6_ESM.pptx]

## Slide 1
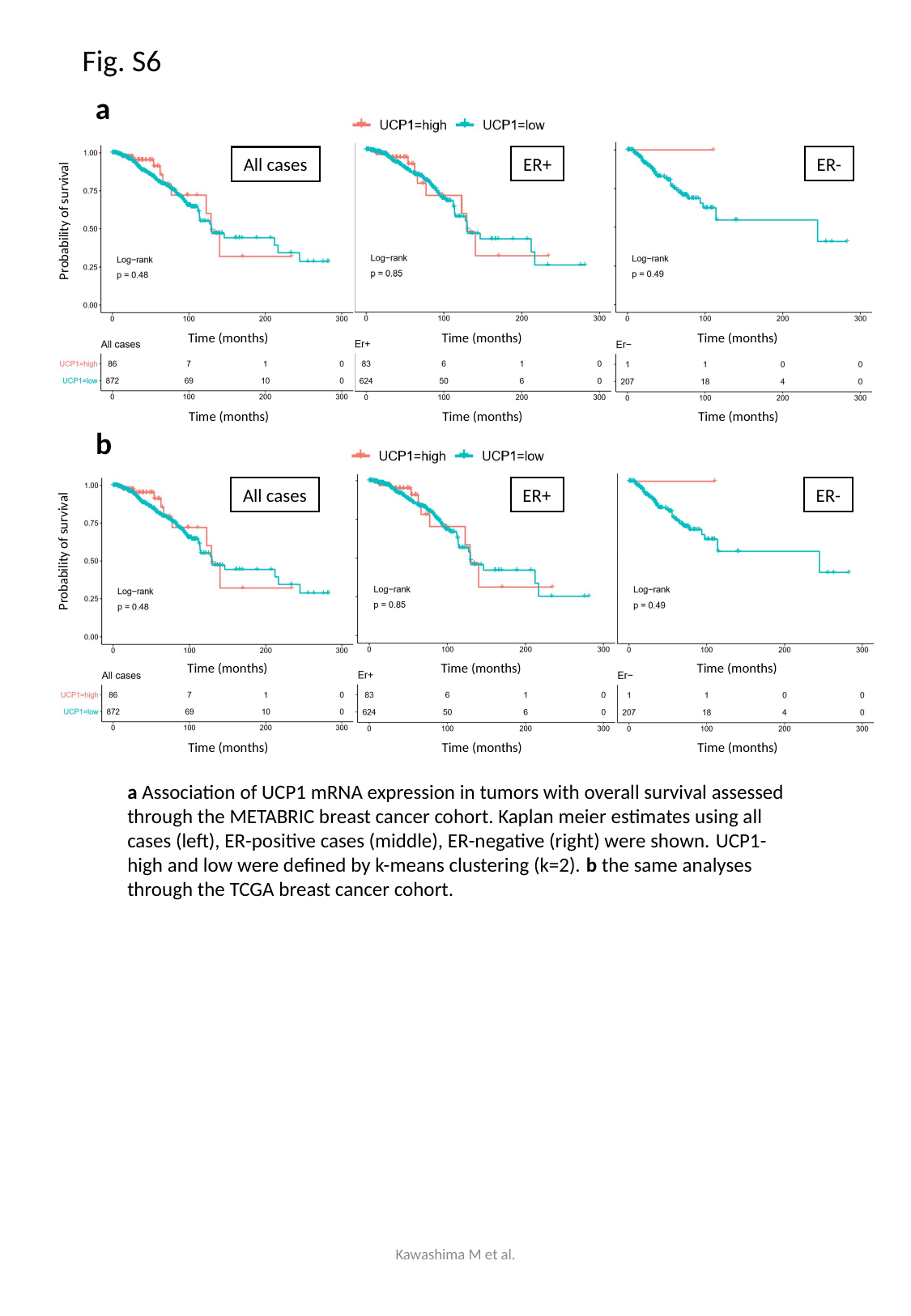

Fig. S6
a
ER+
ER-
All cases
Probability of survival
Time (months)
Time (months)
Time (months)
Time (months)
Time (months)
Time (months)
b
ER+
ER-
All cases
Probability of survival
Time (months)
Time (months)
Time (months)
Time (months)
Time (months)
Time (months)
a Association of UCP1 mRNA expression in tumors with overall survival assessed through the METABRIC breast cancer cohort. Kaplan meier estimates using all cases (left), ER-positive cases (middle), ER-negative (right) were shown. UCP1-high and low were defined by k-means clustering (k=2). b the same analyses through the TCGA breast cancer cohort.
Kawashima M et al.
