# Supplementary figures and images for: Disruption of hypoxia-inducible fatty acid binding protein 7 induces beige fat-like differentiation and thermogenesis in breast cancer cells
Source: Cancer Metab. 2020 Jul 6;8:13. doi: 10.1186/s40170-020-00219-4 (PMC7336487; doi:10.1186/s40170-020-00219-4)

## Slide 1
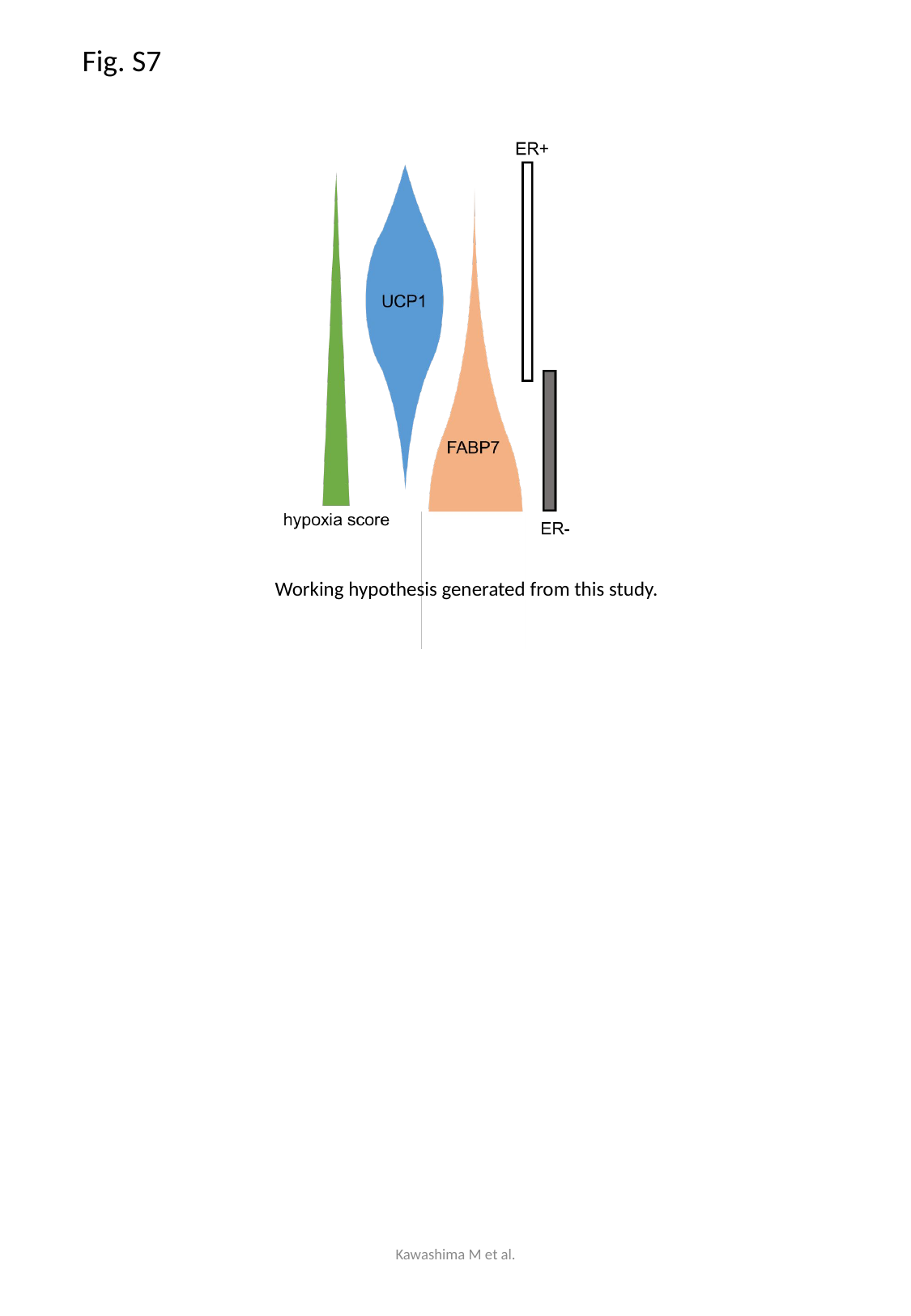

Fig. S7
Working hypothesis generated from this study.
Kawashima M et al.

Supplement: Supplementary file 7 — Additional file 7: Figure S7. Working hypothesis generated from this study. [file 40170_2020_219_MOESM7_ESM.pptx]
